# PI3K/mTOR is a therapeutically targetable genetic dependency in diffuse intrinsic pontine glioma

**DOI:** 10.1172/JCI170329

**Published:** 2024-02-06

**Authors:** Ryan J. Duchatel, Evangeline R. Jackson, Sarah G. Parackal, Dylan Kiltschewskij, Izac J. Findlay, Abdul Mannan, Dilana E. Staudt, Bryce C. Thomas, Zacary P. Germon, Sandra Laternser, Padraic S. Kearney, M. Fairuz B. Jamaluddin, Alicia M. Douglas, Tyrone Beitaki, Holly P. McEwen, Mika L. Persson, Emily A. Hocke, Vaibhav Jain, Michael Aksu, Elizabeth E. Manning, Heather C. Murray, Nicole M. Verrills, Claire Xin Sun, Paul Daniel, Ricardo E. Vilain, David A. Skerrett-Byrne, Brett Nixon, Susan Hua, Charles E. de Bock, Yolanda Colino-Sanguino, Fatima Valdes-Mora, Maria Tsoli, David S. Ziegler, Murray J. Cairns, Eric H. Raabe, Nicholas A. Vitanza, Esther Hulleman, Timothy N. Phoenix, Carl Koschmann, Frank Alvaro, Christopher V. Dayas, Christopher L. Tinkle, Helen Wheeler, James R. Whittle, David D. Eisenstat, Ron Firestein, Sabine Mueller, Santosh Valvi, Jordan R. Hansford, David M. Ashley, Simon G. Gregory, Lindsay B. Kilburn, Javad Nazarian, Jason E. Cain, Matthew D. Dun

**Affiliations:** 1 Cancer Signalling Research Group, School of Biomedical Sciences and Pharmacy, College of Health, Medicine and Wellbeing, University of Newcastle, Callaghan, New South Wales, Australia.; 2 Precision Medicine Research Program, Hunter Medical Research Institute, New Lambton Heights, New South Wales, Australia.; 3 Paediatric Stream, Mark Hughes Foundation Centre for Brain Cancer Research, College of Health, Medicine, and Wellbeing, Callaghan, New South Wales, Australia.; 4 Centre for Cancer Research, Hudson Institute of Medical Research, Clayton, Victoria, Australia.; 5 Department of Molecular and Translational Science, Monash University, Clayton, Victoria, Australia.; 6 School of Biomedical Science and Pharmacy, College of Health, Medicine and Wellbeing, University of Newcastle, Callaghan, New South Wales, Australia.; 7 DIPG/DMG Research Center Zurich, Children’s Research Center, Department of Pediatrics, University Children’s Hospital Zürich, Zurich, Switzerland.; 8 Duke Molecular Physiology Institute, Duke University School of Medicine, Durham, North Carolina, USA.; 9 Infertility and Reproduction Research Program, Hunter Medical Research Institute, New Lambton Heights, New South Wales, Australia.; 10 Children’s Cancer Institute, University of New South Wales (UNSW) Sydney, Kensington, New South Wales, Australia.; 11 School of Clinical Medicine, UNSW Medicine and Health, UNSW Sydney, Kensington, New South Wales, Australia.; 12 Kids Cancer Centre, Sydney Children’s Hospital, Randwick, New South Wales, Australia.; 13 Johns Hopkins University School of Medicine, Baltimore, Maryland, USA.; 14 Ben Towne Center for Childhood Cancer Research, Seattle Children’s Research Institute, Seattle, Washington, USA.; 15 Department of Pediatrics, Seattle Children’s Hospital, University of Washington, Seattle, Washington, USA.; 16 Princess Máxima Center for Pediatric Oncology, Utrecht, Netherlands.; 17 Division of Pharmaceutical Sciences, James L. Winkle College of Pharmacy, University of Cincinnati, Cincinnati, Ohio, USA.; 18 Division of Pediatric Hematology/Oncology, Department of Pediatrics, University of Michigan, Ann Arbor, Michigan, USA.; 19 John Hunter Children’s Hospital, New Lambton Heights, New South Wales, Australia.; 20 Department of Radiation Oncology, St. Jude Children’s Research Hospital, Memphis, Tennessee, USA.; 21 Department of Radiation Oncology Northern Sydney Cancer Centre, Royal North Shore Hospital, St Leonards, New South Wales, Australia.; 22 The Brain Cancer group, St Leonards, New South Wales, Australia.; 23 Sydney Medical School, University of Sydney, Sydney, Australia.; 24 Department of Medical Oncology, Peter MacCallum Cancer Centre, Melbourne, Victoria, Australia.; 25 Personalised Oncology Division, The Walter and Eliza Hall Institute of Medical Research, Parkville, Victoria, Australia.; 26 Department of Medical Biology, University of Melbourne, Parkville, Victoria, Australia.; 27 Children’s Cancer Centre, The Royal Children’s Hospital Melbourne, Parkville, Victoria, Australia.; 28 Neuro-Oncology Laboratory, Murdoch Children’s Research Institute, Department of Paediatrics, University of Melbourne, Parkville, Victoria, Australia.; 29 Department of Neurology, Neurosurgery, and Pediatrics, University of California, San Francisco, California, USA.; 30 Department of Paediatric and Adolescent Oncology/Haematology, Perth Children’s Hospital, Nedlands, Washington, Australia.; 31 Brain Tumour Research Laboratory, Telethon Kids Institute, Nedlands, Washington, Australia.; 32 Division of Paediatrics, University of Western Australia Medical School, Nedlands, Western Australia, Australia.; 33 Michael Rice Centre for Hematology and Oncology, Women’s and Children’s Hospital, North Adelaide, South Australia, Australia.; 34 South Australia Health and Medical Research Institute, Adelaide, South Australia, Australia.; 35 South Australian Immunogenomics Cancer Institute, Faculty of Health and Medical Sciences, The University of Adelaide, Adelaide, South Australia, Australia.; 36 The Preston Robert Tisch Brain Tumor Center at Duke, Department of Neurosurgery, Duke University, Durham, North Carolina, USA.; 37 Center for Genetic Medicine Research, Children’s National Hospital, Washington, DC, USA.; 38 The George Washington University, School of Medicine and Health Sciences, Washington, DC, USA.

**Keywords:** Oncology, Therapeutics, Brain cancer, Drug therapy, Oncogenes

## Abstract

Diffuse midline glioma (DMG), including tumors diagnosed in the brainstem (diffuse intrinsic pontine glioma; DIPG), are uniformly fatal brain tumors that lack effective treatment. Analysis of CRISPR/Cas9 loss-of-function gene deletion screens identified PIK3CA and MTOR as targetable molecular dependencies across patient derived models of DIPG, highlighting the therapeutic potential of the blood-brain barrier–penetrant PI3K/Akt/mTOR inhibitor, paxalisib. At the human-equivalent maximum tolerated dose, mice treated with paxalisib experienced systemic glucose feedback and increased insulin levels commensurate with patients using PI3K inhibitors. To exploit genetic dependence and overcome resistance while maintaining compliance and therapeutic benefit, we combined paxalisib with the antihyperglycemic drug metformin. Metformin restored glucose homeostasis and decreased phosphorylation of the insulin receptor in vivo, a common mechanism of PI3K-inhibitor resistance, extending survival of orthotopic models. DIPG models treated with paxalisib increased calcium-activated PKC signaling. The brain penetrant PKC inhibitor enzastaurin, in combination with paxalisib, synergistically extended the survival of multiple orthotopic patient-derived and immunocompetent syngeneic allograft models; benefits potentiated in combination with metformin and standard-of-care radiotherapy. Therapeutic adaptation was assessed using spatial transcriptomics and ATAC-Seq, identifying changes in myelination and tumor immune microenvironment crosstalk. Collectively, this study has identified what we believe to be a clinically relevant DIPG therapeutic combinational strategy.

## Abstract 2

The brain penetrant pan-PI3K inhibitor paxalisib targets PIK3CA and shows enhanced efficacy with radiotherapy, enzastaurin, and metformin in diffuse intrinsic pontine glioma.

## Graphical abstract

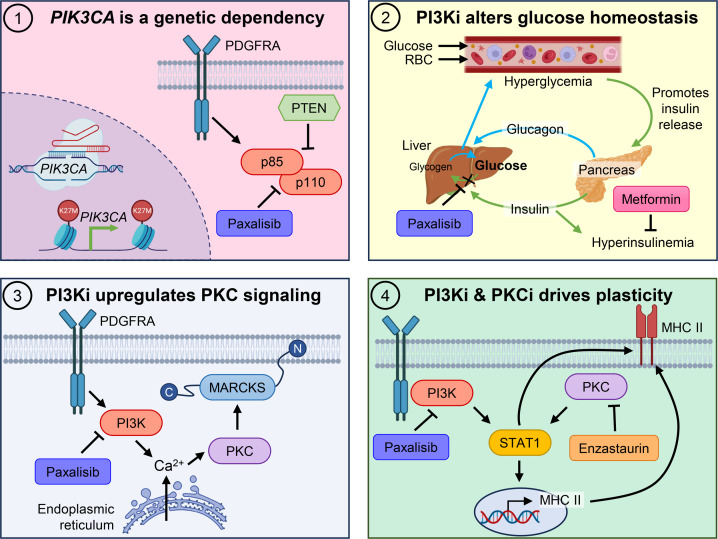

## Introduction

Diffuse midline glioma (DMG), including diffuse intrinsic pontine glioma (DIPG), are fatal high-grade gliomas (HGGs) diagnosed in the midline structures of the brain. DIPG is responsible for more brain tumor-related deaths in children than any other cancer (1). Palliative radiotherapy (RT) is only beneficial for symptom control, with median overall survival of 9–11 months after diagnosis (2, 3).

Global hypomethylation of histone H3 at lysine 27 (H3K27me3) is the molecular hallmark of DIPG, leading to loss of gene silencing, chromatin plasticity, and promotion of prooncogenic transcriptional programs for which there are no approved treatments (4, 5). Global loss of H3K27me3 is driven by recurring mutations (namely, K27M) in histone H3 genes including, *HIST1H3B/C* or *H3F3A* (4, 6), or through overexpression of the EZH inhibitory protein (EZHIP) (7), both of which inhibit the catalysis of H3K27 trimethylation by the polycomb repressive complex 2 (PRC2) (8). The recent World Health Organization’s fifth Classification of CNS Tumors designates DMG as ‘H3 K27-altered,’ indicating that global hypomethylation of H3K27 is seen in all patients with DMG (9). Herein, we use the term DIPG to collectively refer to both H3 WT (including EZHIP overexpression) and H3 K27M–mutant diffuse pontine gliomas. H3-alterations in DIPG are instigating mutations, but are accompanied by partner mutation(s) in signaling genes (*PDGFRA, ACVR1, PIK3CA, PIK3R1, EGFR*), and/or tumor suppressor genes (*TP53, PPM1D, PTEN, BCOR*), with cooccurrence of either necessary to induce malignant growth (10, 11). Cosegregation of discrete components of the PI3K/Akt/mTOR signaling axis are recognized as recurrent molecular drivers of H3 K27-altered gliomas (3), with recurring mutations or amplifications in *PDGFRA* driving constitutive activation of the PI3K signaling axis (12). Activated PI3K/Akt/mTOR signaling drives angiogenesis, cancer cell metabolism, growth, and survival (11), highlighting the potential of therapies that show activity in the CNS and target this oncogenic signaling axis for the treatment of DIPG.

Targeting PI3K/Akt/mTOR has been tested across almost all cancer types (13). Although there are more than 40 different inhibitors in various stages of clinical development, only mTOR inhibitors such as temsirolimus (14) and everolimus (15) and PI3K inhibitors idelalisib and copanlisib (16), have gained FDA approval as anticancer therapies; however, PI3K inhibitors often show limited activity in the CNS.

The CNS penetrant, pan-PI3K/Akt/mTOR (p110α, p110β, p110δ, and p110γ) inhibitor paxalisib (formerly GDC-0084), was developed for the treatment of glioblastoma, as approximately 80% of cases harbor recurring mutations/amplification in genes mapping to the PI3K signaling axis (17). Specifically optimized to cross the blood-brain barrier (BBB) (18), paxalisib has completed dose escalation and maximum tolerated dose (MTD) clinical trials for DIPG, identifying a dose of 27 mg/m^2^/day (NCT03696355) (19), following human trials in adults with recurrent HGG, where paxalisib showed brain penetration at clinically relevant concentrations, with 40% of patients achieving stable disease (20, 21). Importantly, treatment-induced transient hyperinsulinemia is a major driver of reduced efficacy of PI3K/Akt/mTOR inhibitors, promoting glycogen breakdown and inhibition of glucose uptake, resulting in hyperglycemia (22, 23). Hence, patients experience compensatory insulin release from the pancreas to restore normal glucose homeostasis, promoting side effects and activation of insulin feedback pathways that reactivate PI3K/AKT/mTOR signaling in tumors, particularly when continuous PI3K inhibition is attempted in isolation (24).

The complex and heterogeneous somatic, epigenetic, and clonal landscapes of DIPG render monotherapeutic approaches unlikely to promote long-term survival (25, 26). Therefore, combination strategies that synergize and exploit the unique biological features of DIPG are needed. Here, we analyzed a targeted patient-derived DIPG model CRISPR/Cas9 gene deletion data set (27) and identified PI3K/mTOR as genetic dependencies, required for the transmission of oncogenic signals. Furthermore, we have addressed the therapeutic limitations of paxalisib-induced transient hyperinsulinemia using dose optimization alone and in combination with metformin (19). Utilizing a multiomic strategy including transcriptomics and quantitative phosphoproteomics of DIPG cells treated with paxalisib, we have identified increased calcium-induced protein kinase C (PKC) signaling, suggestive of a combined therapeutic vulnerability that we have exploited using the brain penetrant PKC inhibitor enzastaurin. Assessment of the impact of the epigenome and therapeutic adaptability using assay for transposase-accessible chromatin using sequencing (ATAC-Seq) and xenium spatial transcriptomics identified that the combination of paxalisib, metformin, and enzastaurin altered DIPG cell myelination programs and promoted crosstalk with the tumor immune microenvironment (TIME) that may be targetable with additional, or sequential therapies. In this study we address the intrinsic neoplastic sequela of DIPG by combining standard-of-care RT with the targeting of PI3K/Akt/mTOR using paxalisib and compensatory PKC signaling using enzastaurin, coupled with strategies to manage treatment-related side effects using metformin. This is a clinically relevant and feasible combination strategy for the treatment of patients with DIPG to be studied in clinical trials.

## Results

### Integrated CRISPR/Cas9 loss-of-function and drug screening predicts PIK3CA and MTOR to be genetic dependencies in DIPG.

**Figure 1:**
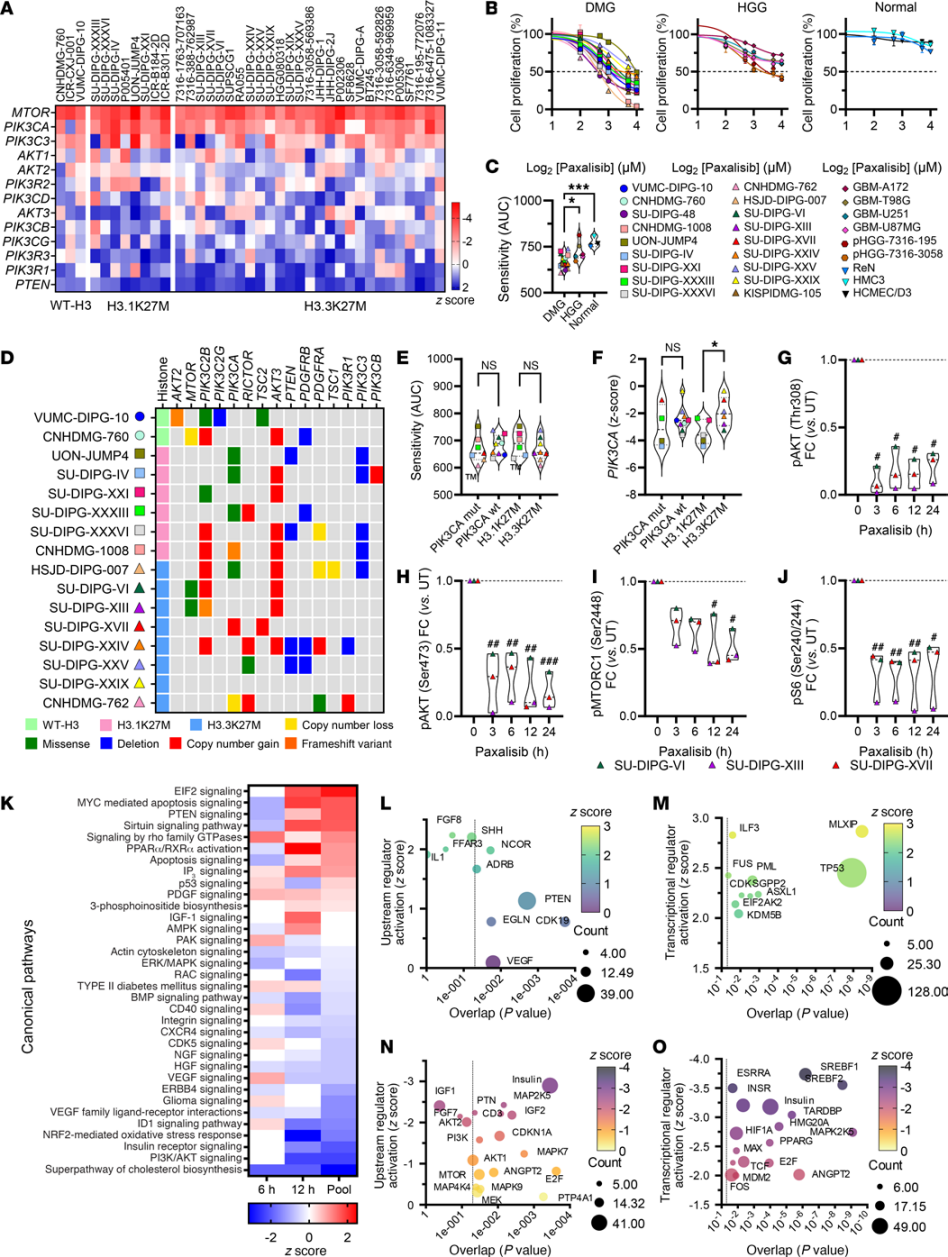
Patient-derived DMG cell lines are sensitive to paxalisib in vitro. (**A**) CRISPR/Cas9 loss-of-function screening across H3K27-altered subtypes of DMG; WT-H3 (EZHIP) (*n* = 3), H3.1K27M (*n* = 8), H3.3K27M (*n* = 27). (**B**) Sensitivity of DMG WT-H3 (circles), H3.1K27M (squares), H3.3K27M (triangles), (*n* = 18), GBM (diamonds) (*n* = 4) and HGG (hexagons) (*n* = 2) patient-derived cell lines and normal (upsidedown triangle) (*n* = 3) to 72 hours paxalisib treatment. (**C**) Comparison of DMG to HGG/GBM and normal cell lines (AUC) to 72 hours paxalisib treatment (DMG versus HGG/GBM, *P* = 0.0023 and normal *P* = 0.0008, 1-way ANOVA). (**D**) Oncoprint of aberrations (TSO500) in DIPG cell lines (*n* = 16). (**E**) Comparison of paxalisib sensitivity (AUC) mutant versus WT *PIK3CA* DMG cell lines and H3K27M mutation subgroups (1-way ANOVA). (**F**) Analysis of paxalisib sensitivity versus *PIK3CA* AUC z-score in *PIK3CA* mutant versus WT DIPG cell lines and H3K27M mutation subgroups (*PIK3CA* mut versus *PIK3CA* WT; H3.1K27M versus H3.3K27M; 1-way ANOVA, *P < 0.05). (**G**–**J**) Phosphorylation of PI3K/Akt/mTOR signaling proteins after 1 μM paxalisib treatment for 3, 6, 12, and 24 hours, SU-DIPG-VI, SU-DIPG-XIII and SU-DIPG-XVII (*n* = 3, 1-way ANOVA, treated versus untreated; ^#^*P* < 0.05, ^##^*P* < 0.01, ^###^*P* < 0.001). Analysis of altered gene expression of SU-DIPG-VI following 6 and 12 hours 1 μM paxalisib treatment identifying (**K**) activated canonical pathways (red) and inactivated pathways (blue); (**L**) upregulated upstream regulators, and (**M**) transcriptional regulators; (**N**) decreased upstream regulators, and (**O**) transcriptional regulators (activation z-score, *P* value, size correlating to number of target molecules in data set).

To determine the importance of the expression of PI3K/Akt/mTOR genes in the transmission of oncogenic signals that promote the growth and proliferation of DIPG, we analyzed a CRISPR/Cas9 loss-of-function screen data set performed on 38 DMG cell lines, representing all DMG H3 K27–altered subtypes (27). Of the 13 genes mapping to the PI3K/Akt/mTOR signaling axis, strong genetic dependency is shown for *PIK3CA* and *MTOR* (Figure 1A). This was confirmed using 2 patient-derived DIPG models that showed significantly diminished proliferation in vitro following knockdown of *PIK3CA* (Supplemental Figure 1A; supplemental material available online with this article; https://doi.org/10.1172/JCI170329DS1). Conversely, knockdown of *PTEN,* the negative regulator of PI3K-signaling, conferred a growth advantage (Figure 1A).

The high frequency and hence evolutionary selection of recurring mutations in *PIK3CA*, *PIK3R1,* and *PTEN* seen in DMG/DIPG (3, 11, 25) provided the impetus to target PI3K-signaling upstream in the signaling cascade rather than downstream targeting of mTOR. Analysis of the Cancer Dependency Map (DepMap) (28) classified *PIK3CA* to be a strongly selective dependency in other cancer types, however, *MTOR* was identified to be a common dependency in both healthy and cancerous cells, providing further justification for targeting PI3K in DIPG. Hence, we profiled proliferation of cells treated with the brain penetrant PI3K/Akt inhibitor paxalisib using DIPG, and nonmidline pediatric HGG cell lines (glioblastoma) (Figure 1B). DIPG cell lines were significantly more sensitive to paxalisib than nonmidline HGGs, with normal controls (HCMEC/D3 BBB endothelial cells, HMC3 microglial cells and ReN neural progenitor cells) resistant to treatment at high doses (Figure 1, B and C). To determine whether recurring somatic alterations influenced sensitivity to paxalisib, representative DIPG cell lines were subjected to next-generation sequencing (NGS) analysis (Figure 1D). Cell line models harbored H3K27M-mutations commensurate with that seen in the population of patients with DIPG (60:30:10, H3.3K27M: H3.1K27M: H3-WT) (3). Thirty percent of the models tested also harbored mutations in *PIK3CA*, 23% carried amplifications or mutations in *MTOR* or *RICTOR*, with the loss of *PTEN* identified in 25% of models (Figure 1D). No significant difference was seen in the sensitivity to paxalisib between DIPG neurosphere cell lines harboring WT or *PIK3CA*-mutations, or between H3.1K27M and H3.3K27M subtypes (Figure 1E).

By combining CRISPR/Cas9 *PIK3CA*–dependency data and NGS data, we aimed to determine the importance of recurring mutations on the level of *PIK3CA* dependency (Figure 1, A and D). These analyses identified no difference in the level of *PIK3CA* dependence comparing *PIK3CA-*mutant versus WT-*PIK3CA–*DIPG models (Figure 1F). H3.1K27M models were significantly more dependent on *PIK3CA* than H3.3K27M models (Figure 1F), with *PIK3CA* mutations more frequently cooccurring with H3.1K27M mutations (25). As activation of the PI3K pathway induces phosphorylation of downstream effector proteins such as Akt and mTOR, we assessed protein expression and phosphorylation to determine whether abundance of pAKT (Thr308/Ser473), pMTORC1 (Ser2448), and pS6 (Ser240/Ser244) correlated with paxalisib sensitivity (Supplemental Figure 1B). Phosphorylation of PI3K proteins was seen across DIPG models; however, the level of phosphorylation did not correlate with in vitro sensitivity to paxalisib (Supplemental Figure 1C). Treatment of DIPG cell line models with paxalisib potently inhibited PI3K/Akt/mTOR phosphorylation, sustained for up to 24 hours after treatment in vitro (Figure 1, G–J and Supplemental Figure 1D).

### Paxalisib treatment modulates insulin and IP3 signaling in DIPG cell lines.

To further our understanding of the anti-DIPG effects of paxalisib treatment, we performed RNA-Seq using the SU-DIPG-VI neurosphere cell line following 6 and 12 hour in vitro paxalisib exposure (IC_50_ Figure 1B and Supplemental Table 2, ST4-S6) and compared the results with DMSO vehicle–treated controls. Analysis of both time points identified a total of 12,285 differentially expressed genes (Supplemental Figure 2A), with 526 significantly downregulated transcripts (4.2%) and 454 significantly upregulated transcripts (3.7%), with key targets validated at the protein level via immunoblotting (Supplemental Figure 2B). Ingenuity Pathway Analysis (IPA) identified PI3K/Akt signaling, the insulin receptor, cholesterol biosynthesis, NRF2-mediated oxidative stress response, MYC, PPAR and EIF2 signaling as the major canonical networks modulated by significant changes in gene expression following paxalisib treatment (Figure 1K). Upstream regulator analysis identified increased PTEN, VEGF, and CDK signaling (Figure 1L) and decreased regulation of insulin signaling (Figure 1M). Intriguingly, given the effect paxalisib has on systemic glucose homeostasis, the major transcription factor predicted to be upregulated by paxalisib treatment was *MLXIP*, the glucose-regulated factor of the Myc/Max/Mad superfamily (Figure 1N). Decreased upstream regulator analysis identified SREBF1/2 transcription factors, which control cholesterol homeostasis by regulating the transcription of sterol-regulated genes (Figure 1O). Altogether, potent modulation of insulin receptor signaling was seen in DIPG cells alongside increased activity of glucose-regulated pathways following paxalisib treatment in vitro (Figure 1, K–O). Indeed, insulin feedback is a well characterized mechanism of resistance to PI3K inhibitors in vivo (22), an important systemic consideration when testing paxalisib against DIPG in vivo.

### Optimized in vivo dosing improved the pharmacokinetic and pharmacodynamic properties of paxalisib in the CNS.

**Figure 2:**
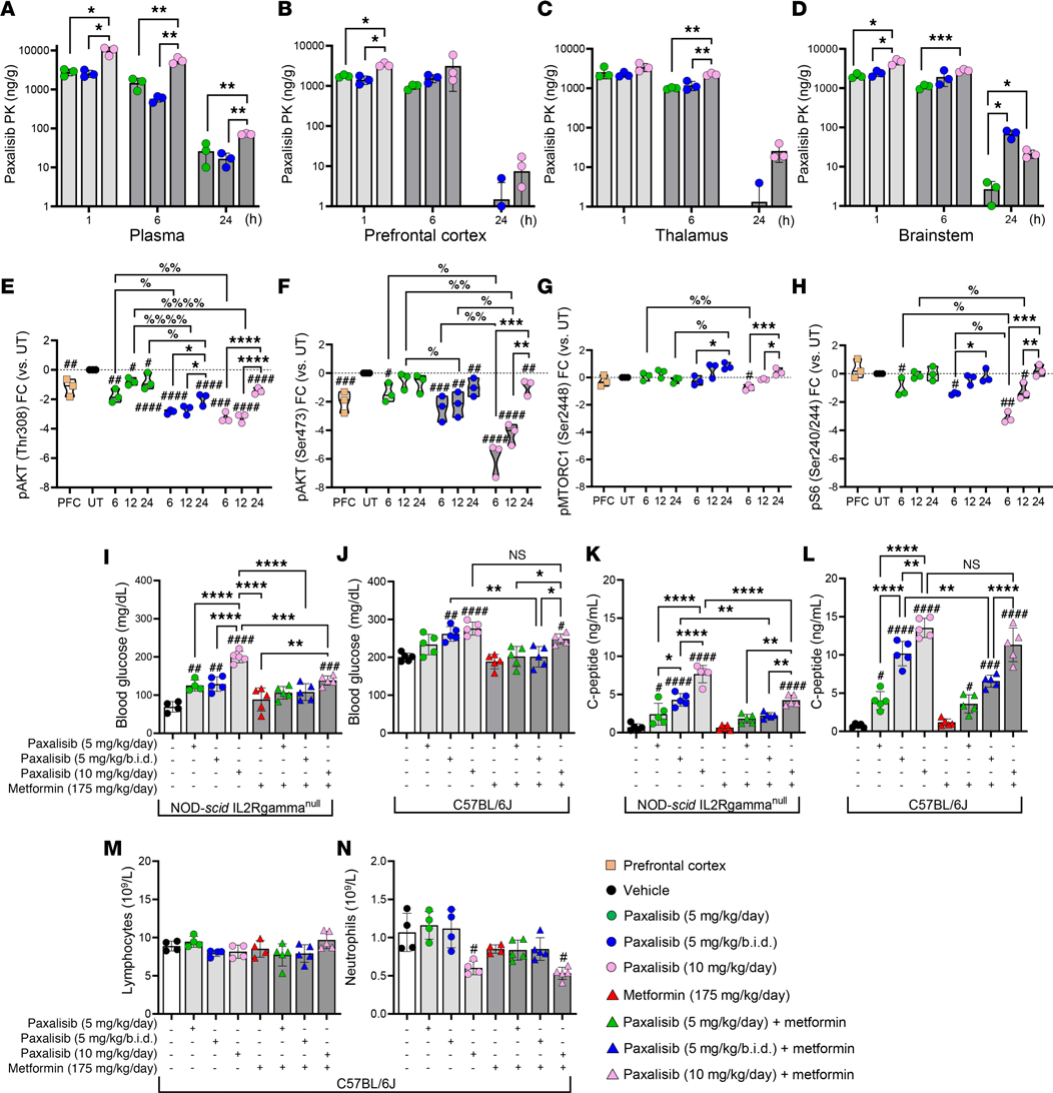
Pharmacokinetics and pharmacodynamics of optimized paxalisib treatment. (**A**–**D**) Paxalisib pharmacokinetics and (**E**–**H**) pharmacodynamics following modified dosing. (**A**–**D**) Concentration of paxalisib in (**A**) plasma, (**B**) prefrontal cortex (PFC), (**C**) thalamus, and (**D**) brainstem, measured by multiple reaction monitoring mass spectroscopy (MRM) ± treatment with paxalisib at 5 mg/kg/day, 5 mg/kg/b.i.d., or 10 mg/kg/day and measured after 1, 6 and 24 hours (1-way ANOVA). (**E**–**H**) Phosphorylation analysis using SU-DIPG-XIII-P* tumor tissue treated with 5 mg/kg/day, 5 mg/kg/b.i.d., or 10 mg/kg/day paxalisib for 2-weeks and resected 6, 12 and 24 hours after treatment (1-way ANOVA). (**I**–**J**) Blood glucose (**K**–**L**) and C-peptide measurements 4 hours after treatment with 5 mg/kg/day, 5 mg/kg/b.i.d., or 10 mg/kg/day paxalisib in combination with 175 mg/kg/day metformin in immunocompromised NSG and immunocompetent C57BL/6J mice. (**M** and **N**) Lymphocyte and neutrophil counts from C57BL/6J mice 4 hours following treatment with 5 mg/kg/day, 5 mg/kg/b.i.d., 10 mg/kg/day paxalisib in combination with 175 mg/kg/day metformin (*n* = 3, 1-way ANOVA, treated versus untreated; ^#^*P* < 0.05, ^##^*P* < 0.01, ^###^*P* < 0.001, ^####^*P* < 0.0001, intra/inter-treatment comparison *^/%^*P* < 0.05, **^/%%^*P* < 0.01, ***^/%%%^*P* < 0.001, ****^/%%%%^P < 0.0001).

Historically, PI3K inhibitors have shown limited benefit for patients with CNS tumors due to their limited capacity to penetrate the BBB. Therefore, increased dosing is required to achieve concentrations sufficient to effectively suppress PI3K-signaling in brain tumors, to promote PI3K-inhibitor–related side effects (rash, mucositis, neutropenia, and hyperglycemia), which reduces patient compliance (29). First-in-human Phase I studies determined a MTD of 45 mg/day paxalisib in the adult recurrent HGG setting, with patients experiencing classical PI3K/mTOR-inhibitor related toxicities (20). These studies showed that at this dose, paxalisib crossed the BBB and had on-target effects. Importantly, oral low dose also showed good brain pharmacokinetic (PK) properties and effective tumor growth inhibition even when used once daily, with the highest concentration of paxalisib in the blood (C-max) reached 2 hours after oral treatment (20). Subsequently, preliminary Phase 1B paxalisib safety and dose escalation studies in children with DIPG determined a MTD of 27 mg/m^2^/day, with patients also experiencing classical PI3K-related toxicities (19). Therefore, to establish the in vivo CNS PK of paxalisib, we treated tumor-naive NSG mice orally using the approximate mouse equivalent of the human MTD (approximately 10 mg/kg/day) (30) and reduced to half-MTD once (5 mg/kg/day) or twice daily (5 mg/kg/b.i.d.) (Figure 2, A–D and Supplemental Figure 3). No significant weight loss was seen across any of the dosing regimens following 2-weeks of treatment (Supplemental Figure 3E). PK analysis showed increased plasma concentrations across all time points using 10 mg/kg/day compared with vehicle and 5 mg/kg once or twice daily (Figure 2A). The observed half-life of paxalisib in the mouse plasma was 6.7 hours for mice treated with 10 mg/kg/day, 2.3 hours for mice treated with 5 mg/kg/day, and 5.8 hours for mice treated with 5 mg/kg/b.i.d., shorter than the 18.7 hour plasma half-life of the drug used in adults at MTD (20).

In brain tissues, increased accumulation of paxalisib was seen using 10 mg/kg/day compared with 5 mg/kg/day, particularly in the prefrontal cortex (Figure 2B) and thalamus (Figure 2C). However, in the brainstem, significantly increased accumulation of paxalisib was seen for mice treated with 5 mg/kg/b.i.d. after 24 hours compared with 5 mg/kg/day, and a nonsignificant increase compared with 10 mg/kg/day (Figure 2D and Supplemental Figure 3, A–D).

To identify PD markers of successful in vivo PI3K/Akt/mTOR inhibition, we engrafted SU-DIPG-XIII-P* patient-derived DIPG cells into the pons of NSG mice. Tumors from SU-DIPG-XIII-P* xenograft mice were resected 28 days after surgery after treatment with an acute dose of paxalisib at either 5 mg/kg/day, 5 mg/kg/b.i.d., or 10 mg/kg/day. In line with in vitro analysis, the phosphorylation of pAKT (Thr308/Ser473) decreased in a dose-dependent manner (Figure 2, E and F and Supplemental Figure 3F). Treatment with 5 mg/kg/b.i.d. maintained suppression of PI3K signaling to a similar level to that of 10 mg/kg/day (Figure 2, E and F and Supplemental Figure 3F). Rebound PI3K signaling was seen after 24 hours; however, to a lesser extent using 5 mg/kg/b.i.d., (Figure 2, E–H and Supplemental Figure 3F) and commensurate with the increased paxalisib accumulation seen in the brainstem of mice at 24 hours using this regimen (Figure 2D). Although 5 mg/kg/b.i.d. significantly decreased phosphorylation of pAKT (Ser473) and phosphorylation of pS6 (Ser240/Ser244) compared with 5 mg/kg/day, 10 mg/kg/day was more effective than both reduced dosing regimens (Figure 2H and Supplemental Figure 3F). Together these data highlight that paxalisib 5 mg/kg/b.i.d. maintains sufficient PK to suppress PI3K/Akt signaling in DMG tumors in vivo compared with 10 mg/kg/day and may decrease classical PI3K-related toxicities while maintaining on-target effects.

### Treatment with paxalisib at MTD promoted hyperinsulinemia/hyperglycemia, which was reduced using half-MTD twice daily alone and in combination with metformin.

Grade 3 hyperglycemia was reported as the only dose limiting toxicity (DLT) for children with DIPG treated with paxalisib at 27 mg/m^2^ (19). Most frequently, grade 3 adverse events at MTD were rash (45%), neutropenia (36%), and hyperglycemia (20%), with the observed half-life of paxalisib in the plasma determined at 20.6 ± 9 hours in children with DIPG (19), similar to adult studies (20). To address the reported hyperinsulinemia/hyperglycemia seen in clinical studies, tumor-naive, immunocompromised NSG and immunocompetent C57BL/6J mice were treated with modified paxalisib dosing regimens, alone and in combination with metformin (a commonly prescribed therapy for type 2 diabetes, used to control blood glucose), daily for 2-weeks (5 days on, 2 days off), and sacrificed 4 hours after final treatment to assess fasting blood glucose and C-peptide levels (surrogate measure of insulin levels). Consistent with previous results of patients treated with PI3K inhibitors, mice treated with the human-equivalent MTD of paxalisib (10 mg/kg/day) experienced significantly elevated blood glucose levels (Figure 2, I and J) and increased C-peptide levels 4 hours after treatment (Figure 2, K and L) in both NSG (blood glucose = 199.8 mg/dL, *P* < *0.0001*, C-peptide = 7.98 ng/mL, *P* < *0.0001*) and C57BL/6J (blood glucose = 273.6 mg/dL, *P* < *0.0001*, C-peptide = 13.28 ng/mL, *P* < *0.0001*) mice. NSG mice treated with paxalisib at lower doses (5 mg/kg/day and 5 mg/kg/b.i.d.) still experienced increased blood glucose (5 mg/kg/day = 120.6 mg/dL, *P* < *0.01* and 5 mg/kg/b.i.d. = 124.2 mg/dL, *P* < *0.01*) and C-peptide levels (5 mg/kg/day = 2.0ng/mL, *P* < *0.05* and 5 mg/kg/b.i.d. = 4.14 ng/mL, *P* < *0.0001*) compared with vehicle controls (blood glucose = 71.10 mg/dL, C-peptide = 0.66 ng/mL); however, blood glucose levels were significantly lower than in mice treated with 10 mg/kg/day (Figure 2, I and K). This response was less pronounced in C57BL/6J mice, where no difference was seen in blood glucose levels between 10 mg/kg/day and either 5 mg/kg/day or 5 mg/kg/b.i.d. paxalisib dosing; however, both were significantly elevated compared with the vehicle (Figure 2J). Although NSG or C57BL/6J mice treated with metformin reduced blood glucose (Figure 2, I and K) and C-peptide levels (Figure 2, J and L) following treatment with 10 mg/kg/day paxalisib, C-peptide levels did not return to baseline. Treatment with metformin in NSG mice decreased C-peptides to levels comparable to vehicle controls in both 5 mg/kg regimens (Figure 2K), but not in C57BL/6J mice (Figure 2L), in which it was decreased but remained elevated compared with controls. Considering, paxalisib-treated C57BL/6J mice harbored significantly elevated baseline blood glucose levels compared with NSG mice, corroborating previous studies that identified that the immune system plays an important role in the regulation of blood glucose homeostasis (31), we examined the effects of modified paxalisib dosing in combination with metformin on key white blood cell populations, including lymphocytes and neutrophils using C57BL/6J mice (Figure 2, M and N). Treatment with 5 mg/kg/day, 5 mg/kg/b.i.d., or 10 mg/kg/day paxalisib did not reduce the number of circulating healthy lymphocytes (Figure 2M), nor did the addition of metformin. However, 10 mg/kg/day paxalisib alone and in combination with metformin decreased total neutrophil counts (Figure 2N), which were not affected by the other lower dosing regimens, including 5 mg/kg/day or 5 mg/kg/b.i.d. ± metformin. These data provide additional preclinical evidence that high-dose paxalisib has immunomodulatory effects and may play a role in reduced patient compliance.

**Figure 3:**
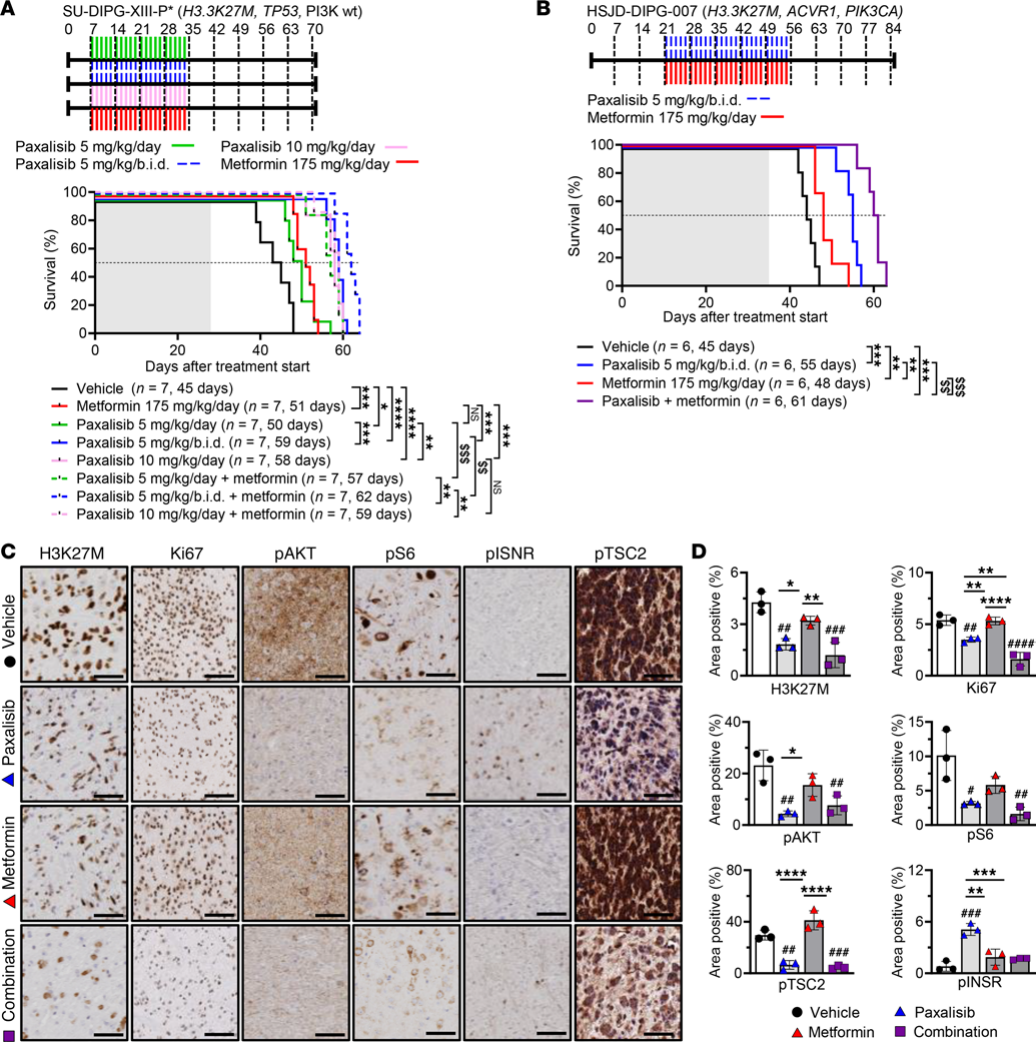
Patient-derived DIPG xenograft model efficacy using optimized paxalisib treatment. (**A**) Kaplan-Meier survival analysis of SU-DIPG-XIII-P* xenografts treated with vehicle, paxalisib 5 mg/kg/day, 5 mg/kg/b.i.d., or 10 mg/kg/day or in combination with metformin 175 mg/kg/day (log-rank test). (**B**) HSJD-DIPG-007 xenografts treated with vehicle, paxalisib 5 mg/kg/b.i.d., metformin 175 mg/kg/day, or combined paxalisib and metformin (log-rank test). Shaded area indicates time receiving treatment. (**C**) Tumor tissue resected from HSJD-DIPG-007 xenografts following 4 weeks of treatment and analyzed by IHC (*n* = 3 mice per treatment, representative images are presented; scale bar: 50 μM) and (**D**) IHC quantified (*n* = 3, 1-way ANOVA, treated versus untreated; ^#^*P* < 0.05, ^##^*P* < 0.01, ^###^*P* < 0.001, ^####^*P* < 0.0001, intra/inter-treatment comparison; *P < 0.05, **P < 0.01, ***P < 0.001, ****P < 0.0001, synergistic comparisons ^$$^*P* < 0.01, ^$$$^*P* < 0.001, shaded area indicates treatment time).

To assess the anti-DIPG efficacy of optimized paxalisib dosing alone and/or in combination with metformin, we employed the SU-DIPG-XIII-P* pontine orthotopic xenograft model (Figure 3A). Encouragingly, 5 mg/kg/day, 5 mg/kg/b.i.d., 10 mg/kg/day paxalisib, and metformin 175 mg/kg/day significantly increased survival compared with the vehicle controls. Furthermore, 5 mg/kg/b.i.d. and 10 mg/kg/day paxalisib significantly extended survival compared with 5 mg/kg/day. Metformin further potentiated the survival benefit of paxalisib 5 mg/kg/day and 5 mg/kg/b.i.d.; however, it did not provide additional benefit to the MTD 10 mg/kg/day regimen, highlighting that more frequent administration of paxalisib at lower dose in combination with metformin may provide the greatest clinical benefit for patients with DIPG. We validated this finding using the PI3K-mutant patient-derived DIPG model HSJD-DIPG-007 (H3.3K27M, *PIK3CA-, ACVR1-*mutant). Again, this optimized regimen significantly extended survival compared with the vehicle, with the combination of paxalisib and metformin synergistically extending survival compared with all treatments (Figure 3B).

IHC analysis identified that paxalisib treatment decreased phosphorylation of Akt and S6 in vivo (Figure 3, C and D). However, using 5 mg/kg/b.i.d. paxalisib alone, phosphorylation of the insulin receptor (INSR) was seen in vivo (Figure 3D), commensurate with the elevated C-peptide levels (Figure 2K). The increased activity of the insulin pathway promoted by paxalisib treatment in DIPG xenograft mouse models was rescued using metformin (Figure 3, C and D), a strategy that effectively dephosphorylated the INSR, promoted increased phosphorylation of tumor suppressor TSC2 at Thr1462, and reduced tumor burden as measured by H3K27M^+^ and Ki67^+^ cells (Figure 3, C and D).

### Paxalisib treatment promotes PKC signaling.

**Figure 4:**
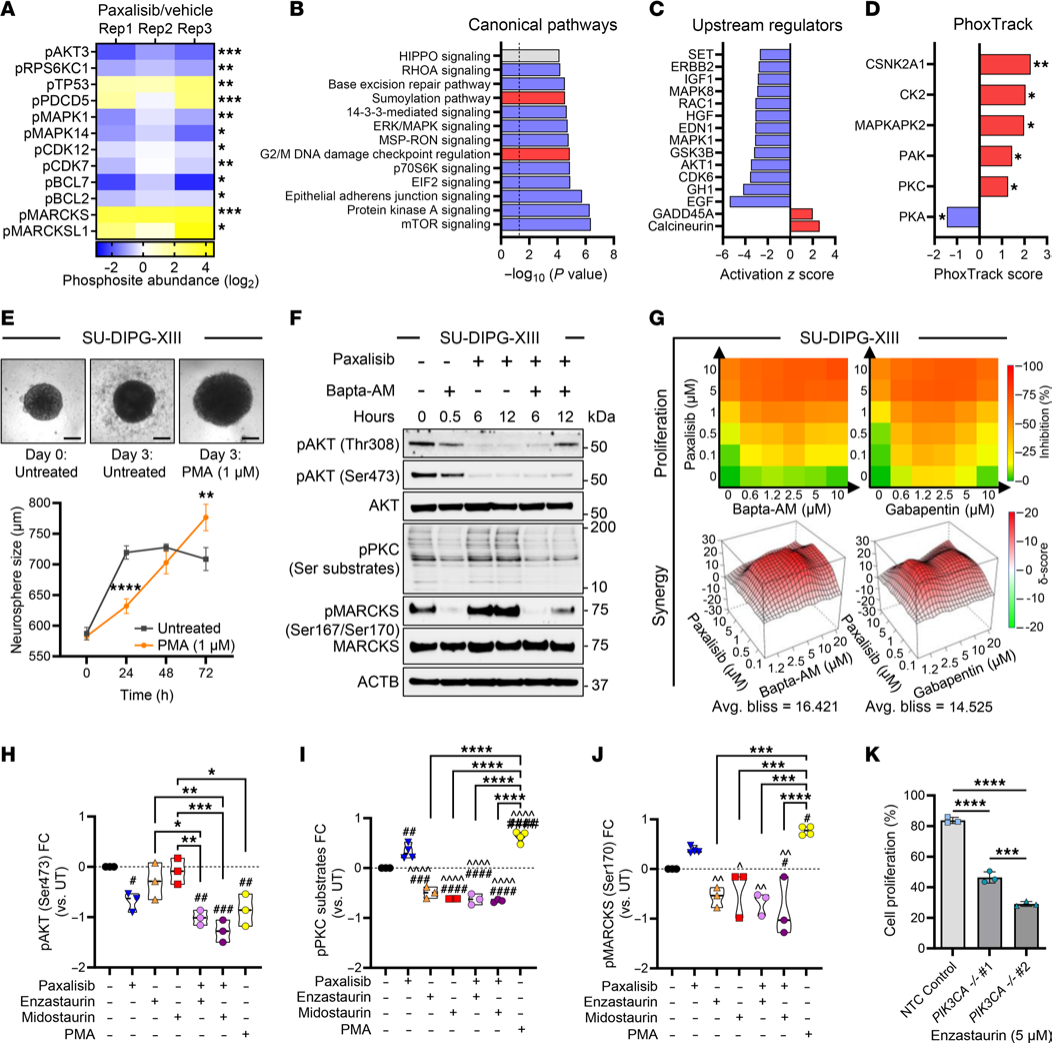
Phosphoproteomic analysis identified potent PKC activation following PI3K inhibition. (**A**) Significantly regulated phosphoproteins following paxalisib treatment (Student’s *t* test; *P < 0.05, **P < 0.01, ***P < 0.001). (**B**) Canonical pathways and (**C**) upstream regulators significantly altered by paxalisib treatment determined by Ingenuity Pathway Analysis (IPA, activated pathways positive z-score (red), inactivated pathways negative z-score (blue)). (**D**) PhoxTrack predicted activated (red), inactivated (blue). (**E**) PKC activated using Phorbol-12-myristate-13-acetate (PMA) using SU-DIPG-XIII cells (scale bar: 200 μM, 2-way ANOVA, PMA versus untreated **P < *0.01*, ****P < *0.0001*). (**F**) BAPTA-AM inhibition of paxalisib-induced PKC substrates and MARCKS phosphorylation, measured by immunoblotting (*n* = 3, representative immunoblot presented). (**G**) Bliss-synergy analysis of the combination of paxalisib with BAPTA-AM and Gabapentin. (**H**–**J**) Quantification of signaling protein phosphorylation following combinations of paxalisib and PKC inhibitors after 24 hours (*n* = 3 biological replicates, 1-way ANOVA, treated versus untreated; ^#^*P* < 0.05, ^##^*P* < 0.01, ^###^*P* < 0.001, ^####^*P* < 0.0001, intra-treatment comparison; *P < 0.05, **P < 0.01, ***P < 0.001, ****P < 0.0001, treated versus paxalisib; ^*P* < 0.05, ^^*P* < 0.01, ^^^^*P* < 0.0001). (**K**) Proliferation of SU-DIPGXXXVI following CRISPR/Cas9 knockdown of *PIK3CA* in cell lines compared with nontargeting control (NTC) and treated with enzastaurin for 72 hours (biological triplicate, 1-way ANOVA; ***P < 0.001, ****P < 0.0001).

To complement and extend the mechanistic insights established by RNA-Seq (Figure 1, K–O) and to garnish a view on the posttranslational landscapes of DIPG following paxalisib treatment, we performed global unbiased quantitative phosphoproteomic profiling of DIPG cells (32–34) treated for 6 hours with paxalisib (IC_50_ paxalisib, Figure 1B and Supplemental Table 7). A total of 6,017 unique proteins and 2,623 unique phosphoproteins were quantitatively identified across samples (Supplemental Figure 4A), with 753 significantly downregulated phosphoproteins and 95 significantly upregulated phosphoproteins (Supplemental Figure 4B). These analyzes further confirmed paxalisib to be a potent inhibitor of the PI3K/Akt/mTOR pathway, while simultaneously increasing phosphorylation of MARCKS (Ser170) and MARCKSL1 (Ser167/S170), both substrates of active PKC signaling (Figure 4A). Kinases modulated in response to paxalisib were identified using Integrative Inferred Kinase Activity (INKA) analysis (35) (Supplemental Figure 4C) and interrogated using IPA, which identified networks and canonical pathways mapping to GSK3B, mTOR, and P70S6K, all regulated by PI3K (Figure 4B), and upstream regulators including AKT1, IGF1, and EGF (Figure 4C). PhoxTrack kinase activation analysis (36) identified kinases significantly upregulated by paxalisib treatment, including CSNK2A1, CK2, MAPKAPK2, PAK, and PKC signaling; these kinases either regulate intracellular calcium release or are influenced by calcium directly (Figure 4D). IPA analysis of RNA-Seq data predicted a significant increase in IP_3_ signaling after paxalisib exposure (Figure 1K and Supplemental Figure 2B). IP_3_ is made through the hydrolysis of phosphatidylinositol 4,5-bisphosphate (PIP2), where it binds to its receptor, IP_3_R1, on the endoplasmic reticulum recycling and releasing calcium (Ca^2+^) into the cytoplasm (37).

Ca^2+^ plays a fundamental role in neuronal plasticity through the regulation of PKC signaling (38). Thus, we evaluated the effect of the PKC activator Phorbol 12-myristate 13-acetate (PMA) on DIPG cells. Exogeneous activation of PKC using PMA significantly increased the DIPG neurosphere growth compared with untreated controls (Figure 4E). Paxalisib treatment of DIPG models increased the phosphorylation of PKC substrates and MARCKS (Ser170), which were ablated using the Ca^2+^ chelator BAPTA-AM (Figure 4F and Supplemental Figure 5), suggesting that Ca^2+^ promotes PKC signaling in response to PI3K inhibition. Next, we assessed cytotoxicity using Ca^2+^ targeting compounds, including BAPTA-AM and the voltage-gated Ca^2+^ ion channel inhibitor, gabapentin. Both were found to be synergistic when combined with paxalisib (Figure 4G). Combining paxalisib with PKC inhibitors, including enzastaurin and midostaurin, potently inhibited AKT signaling (Figure 4H and Supplemental Figure 6), suppressed PKC substrate phosphorylation (Figure 4I) and MARCKS phosphorylation (Figure 4J and Supplemental Figure 6) that was previously promoted by PI3K/Akt inhibition (Figure 4, A, I, and J). Indeed, SU-DIPG-XXXVI cells harboring molecular knockdown of *PIK3CA* were further sensitized to enzastaurin treatment, corroborating the link between PI3K inhibition and PKC activation (Figure 4K, NTC versus *PIK3CA^–/+^ #*1, *P* < 0.0001, NTC versus *PIK3CA^–/+^* #2, *P* < 0.0001). Altogether, the use of Ca^2+^ chelators/channel blockers or PKC inhibitors has the potential to suppress paxalisib-induced PKC activation and could be used as combination strategy to potentiate paxalisib efficacy.

### High-throughput drug screening confirms the preclinical utility of targets predicted by phosphoproteomic profiling.

**Figure 5:**
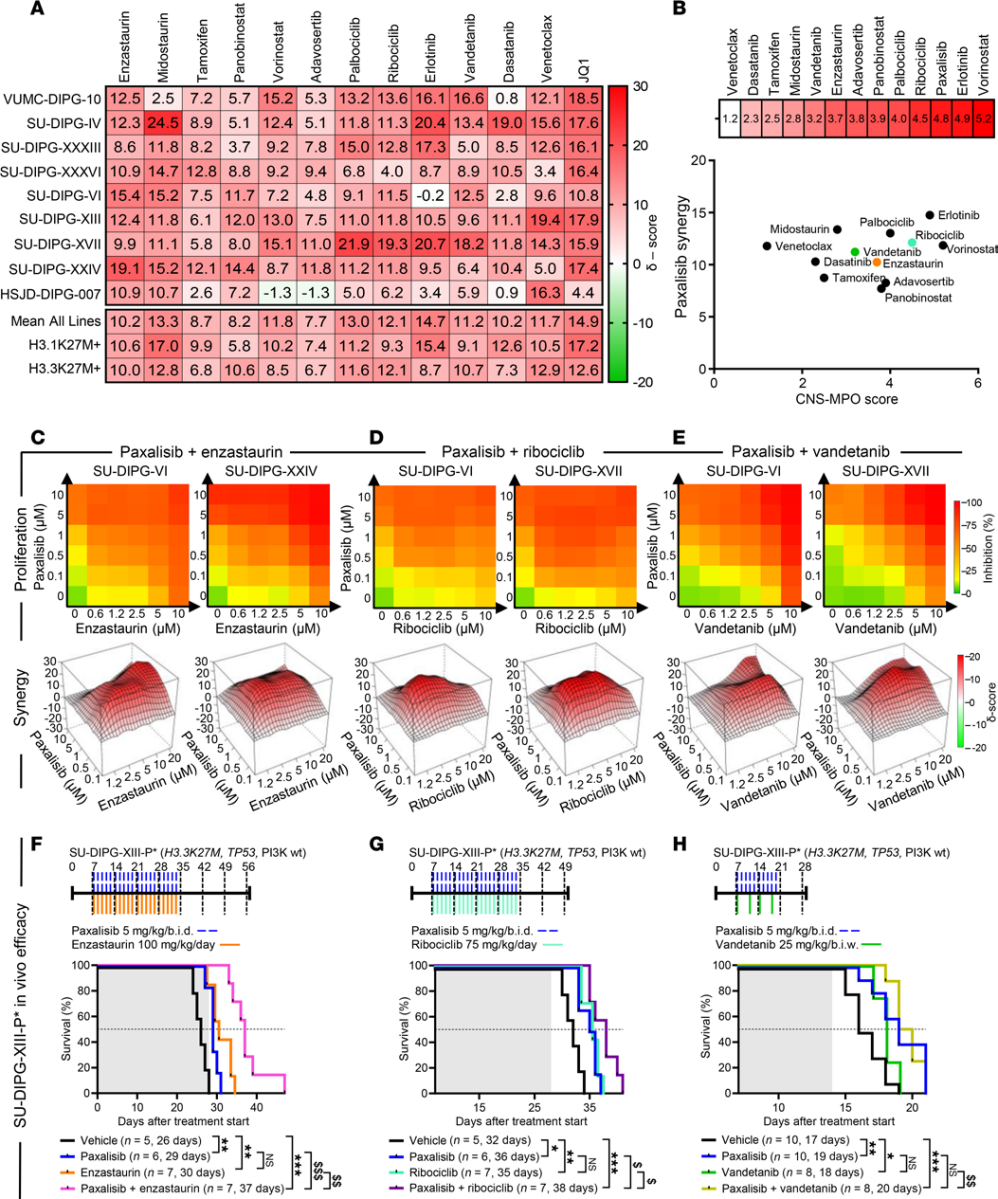
High-throughput drug screen identifies synergistic paxalisib drug combinations. (**A**) Bliss synergy analysis using paxalisib in combination with clinically relevant inhibitors in a panel of DIPG cells lines (*n* = 9), measured by resazurin cell growth and proliferation assays after 72 hours exposure (biological triplicate). (**B**) CNS-MPO analysis of compounds targeting pathways modulated by paxalisib treatment and plotted against paxalisib combination synergy scores. Cell proliferation and bliss synergy analysis for the combination of (**C**) paxalisib and enzastaurin, (**D**) paxalisib and ribociclib, and (**E**) paxalisib and vandetanib. (**F**–**H**) Kaplan-Meier survival analysis of SU-DIPG-XIII-P* xenografts treated with paxalisib (5 mg/kg/b.i.d.) and (**F**) enzastaurin (100 mg/kg/day), (**G**) ribociclib (75 mg/kg/day) and (**H**) vandetanib (25 mg/kg/b.i.w.) (log-rank test, *P < 0.05, **P < 0.01, ***P < 0.001, ****P < 0.0001, synergistic comparisons; ^$^*P* < 0.01, ^$$^*P* < 0.01, ^$$$^*P* < 0.001, shaded area indicates treatment time).

Except in the case of ONC201 (dordaviprone), a small molecule agonist of the mitochondrial protease ClpP (39, 40), beneficial in early phase trials (41) and alternative access opportunities (42–44), monotherapies have unequivocally failed patients with DIPG (25, 26). To identify potential combination strategies, we performed high-throughput combination drug screening assays across a panel of DIPG cell lines (*n* = 9) using paxalisib as a backbone, combined with clinically relevant compound targeting genes (Figure 1, K–O), and/or signaling pathways (Figure 4, A–D) identified via RNA-Seq and/or phosphoproteomic profiling. High-level synergy was seen using the combination of paxalisib and CDK (ribociclib, palbociclib), EGFR, VEGFR (erlotinib and vandetanib), and PKC inhibitors (midostaurin and enzastaurin) (Figure 5A). To identify the best strategy to test in orthotopic DIPG models, we assessed the potential of each drug to penetrate the brain using CNS multi-parameter optimization (MPO) (45) and correlated predicted CNS penetration with paxalisib synergism. These analyzes identified erlotinib, vorinostat, ribociclib, enzastaurin, palbociclib, and vandetanib as potential paxalisib combination strategies (Figure 5B).

Enzastaurin (Figure 5C), ribociclib (Figure 5D), and vandetanib (Figure 5E) are brain-penetrant drugs FDA approved for other indications, which had previously been tested in clinical trials as monotherapies for children diagnosed with DIPG with known MTD and toxicity profiles (46–48) and were elected to test their preclinical efficacy in combination with paxalisib. Enzastaurin (100 mg/kg/day), ribociclib (75 mg/kg/day), vandetanib (25 mg/kg/b.i.w.), and paxalisib (5 mg/kg/b.i.d.) increased survival compared with vehicle controls (Figure 5, F–H). However, only the combination of paxalisib and enzastaurin synergistically enhanced survival compared with the monotherapies (Figure 5F and Supplemental Figure 7A). The combination of paxalisib and ribociclib provided an additive survival benefit (Figure 5G and Supplemental Figure 7B), while the combination of paxalisib and vandetanib provided no additional benefit, potentially due to increased toxicity while administrating both therapies orally, which necessitated a reduced treatment time (Figure 5H and Supplemental Figure 7C).

### Paxalisib and enzastaurin is an effective combination strategy in the upfront setting.

**Figure 6:**
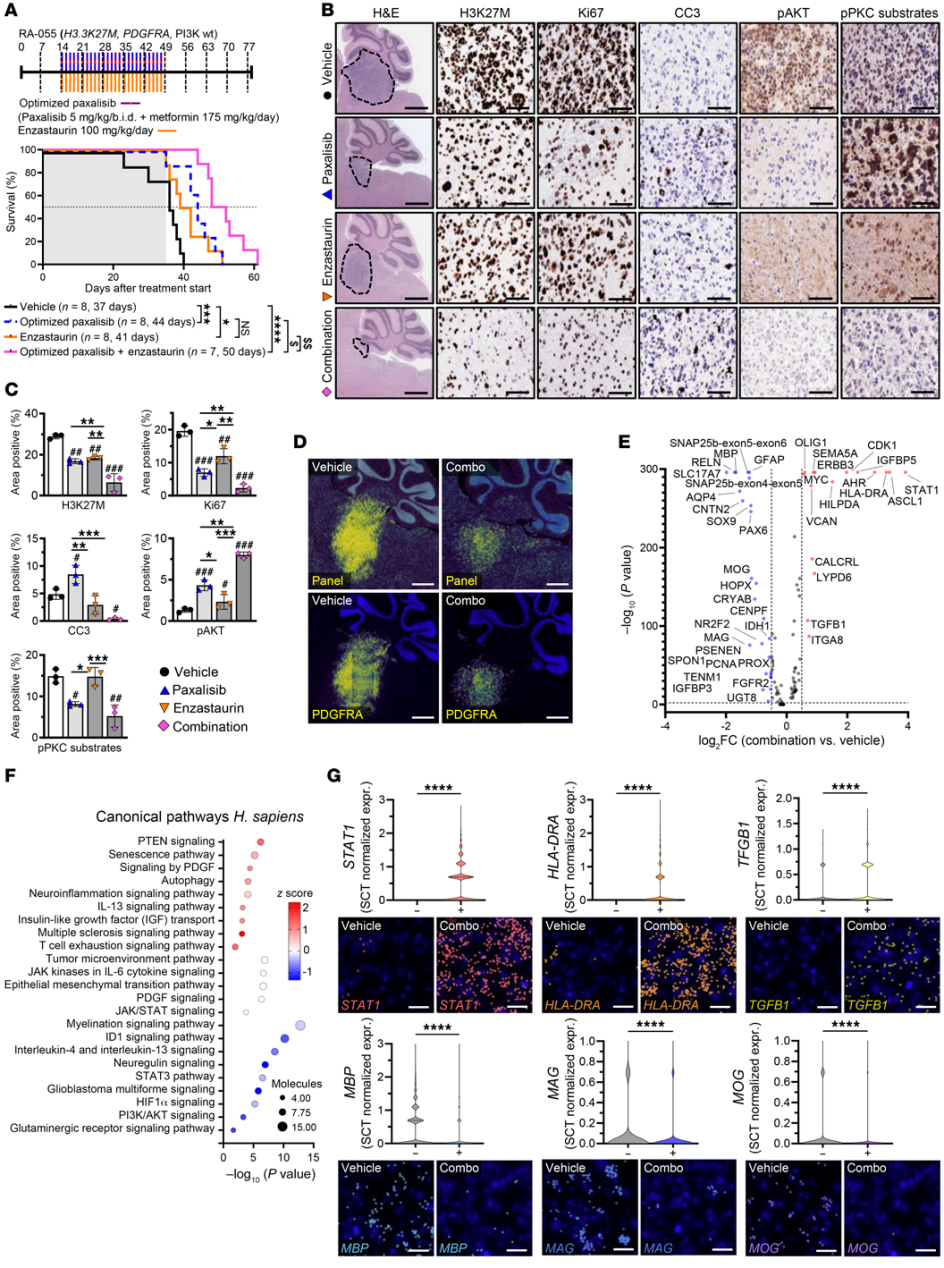
In vivo spatial transcriptomics identifies pathways underpinning therapeutic adaptation. (**A**) Kaplan-Meier survival analysis of RA-055 xenografts treated with the optimized combination of paxalisib (5 mg/kg/b.i.d.) + metformin (175 mg/kg/day) and enzastaurin (100 mg/kg/day) (shaded area indicates treatment time, log-rank test, treated versus untreated; *P < 0.05, **P < 0.01, ***P < 0.001,****P < 0.0001, synergistic comparisons; ^$$^*P* < 0.01, ^$$$^*P* < 0.001). (**B**) Tumor tissue was resected from RA-055 xenografts following 4 weeks of treatment and analyzed by IHC (*n* = 3 per treatment, representative images shown, scale bar: 50 μm) and (**C**) images quantified using ImageJ (technical triplicate, across biological replicates, *n* = 3, 1-way ANOVA, treated versus untreated; *P < 0.05, **P < 0.01, ***P < 0.001, intra-treatment comparison; ^#^*P* < 0.05, ^##^*P* < 0.01, ^###^*P* < 0.001). (**D**) Representative images of 10 × Xenium analysis using a panel of 358 genes, with tumors identified by high *PDGFRA* expression (scale bar: 1,000 μm). (**E**) Differential gene expression analysis (Wilcoxon test) on normalized count data, presented as log_2_FC. (**F**) IPA pathway analysis of significantly altered genes following treatment. (**G**) Significantly altered gene transcripts, *STAT1*, *HLA-DRA*, *TGFB1*, and *MBP*, *MAG*, *MOG* visualized using Xenium Explorer, with corresponding violin plots of SCTransform normalized count data (scale bar: 25 μm, 1-way ANOVA, treated versus untreated; ***P < 0.001, ****P < 0.0001).

Given the encouraging paxalisib and enzastaurin combination results, we validated its survival benefit using the RA-055 DIPG model (biopsy after radiation, DIPG model (49)), and in combination with 175 mg/kg/day metformin, hereafter referred to as “optimized paxalisib”. This combination showed synergy compared with enzastaurin alone and provided an additive benefit compared with optimized paxalisib (Figure 6A). Mice remained symptom free while on the combination, however, they succumbed either due to neurological symptoms or weight loss at the end of treatment (Figure 6A and Supplemental Figure 8, A and B). IHC analysis of tumors resected at the end of treatment (Figure 6, B and C) mirrored the survival benefit, with optimized paxalisib and enzastaurin decreasing tumor burden (H3K27M and Ki67), potentiated by the combination. Indeed, the combination induced in vivo cytotoxicity with increased abundance of apoptotic markers (Figure 6, B and C). Mechanistically, paxalisib decreased pAKT and subsequently promoted PKC signaling commensurate with our in vitro results and was rescued using enzastaurin (Figure 6, B and C).

Although our optimized combination increased overall survival (OS) of our models, mice still succumbed of DIPG, suggesting that the high level of chromatin plasticity characterizing DIPG promotes therapeutic adaptation. Thus, therapeutic escape/plasticity was assessed via single cell spatial transcriptomics analysis (scSTA) using the 10× Genomics Xenium platform (Figure 6, D–G and Supplemental Table 3). Tumor regions were selected for scSTA based on high expression of *PDGFRA* (Figure 6D). Across the 358 human glioma gene panel, differential-expression scSTA identified 16 significantly upregulated and 26 downregulated genes in response to the combination (Figure 6E). Increased expression of *STAT1* was seen following treatment with optimized paxalisib and enzastaurin alone, potentiated by the combination (Figure 6G and Supplemental Table 8). Increased STAT1 signaling and decreased *RELN* expression (50) (Figure 6E and Supplemental Table 8) potentially underpinned the increased *TGFB1* and MHC II (*HLA-DRA*) gene expression profiles identified following 4 weeks of treatment (Figure 6, E–G and Supplemental Table 8). Induction of expression of cell surface MHC I and MHC II has previously been shown in head and neck squamous cell carcinomas following PI3K inhibition (51) and in *PIK3CA* mutant human bladder cancers treated with the PI3K inhibitor BKM120 (52). In these studies, BKM120 increased IFN-γ to promote STAT1 protein expression levels, supporting our in vivo DIPG results treated with the combination (Figure 6, E–G). This suggests that DIPG’s genetic dependence on PI3K signaling may promote immune escape via effects on antigen presentation, highlighting the need for this combination to be explored in immunocompetent mouse models. Genes critical to oligodendroglial myelination, including myelin-associated glycoprotein (*MAG*), myelin basic protein (*MBP*), and myelin oligodendrocyte glycoprotein (*MOG*), showed significantly decreased expression in tumor tissue following 4-week treatment with the combination (Figure 6, E–G and Supplemental Table 8).

Pathway analysis of genes significantly altered by the combination identified upregulation of *PTEN* in line with PI3K inhibition, with a commensurate increase in PDGF signaling, linked to activation of the JAK/STAT signaling in compensation for the loss of PKC and PI3K activity following 4 weeks of treatment (Figure 6F). As our optimized combination strategy decreased PI3K/Akt and neuregulin signaling (a consequence of PKC inhibition), reduced glioblastoma signaling was predicted corresponding to the reduced expression of *GFAP* (Figure 6E, F and Supplemental Table 8).

### Optimized paxalisib and enzastaurin treatment provided a survival benefit in the advanced disease setting.

**Figure 7:**
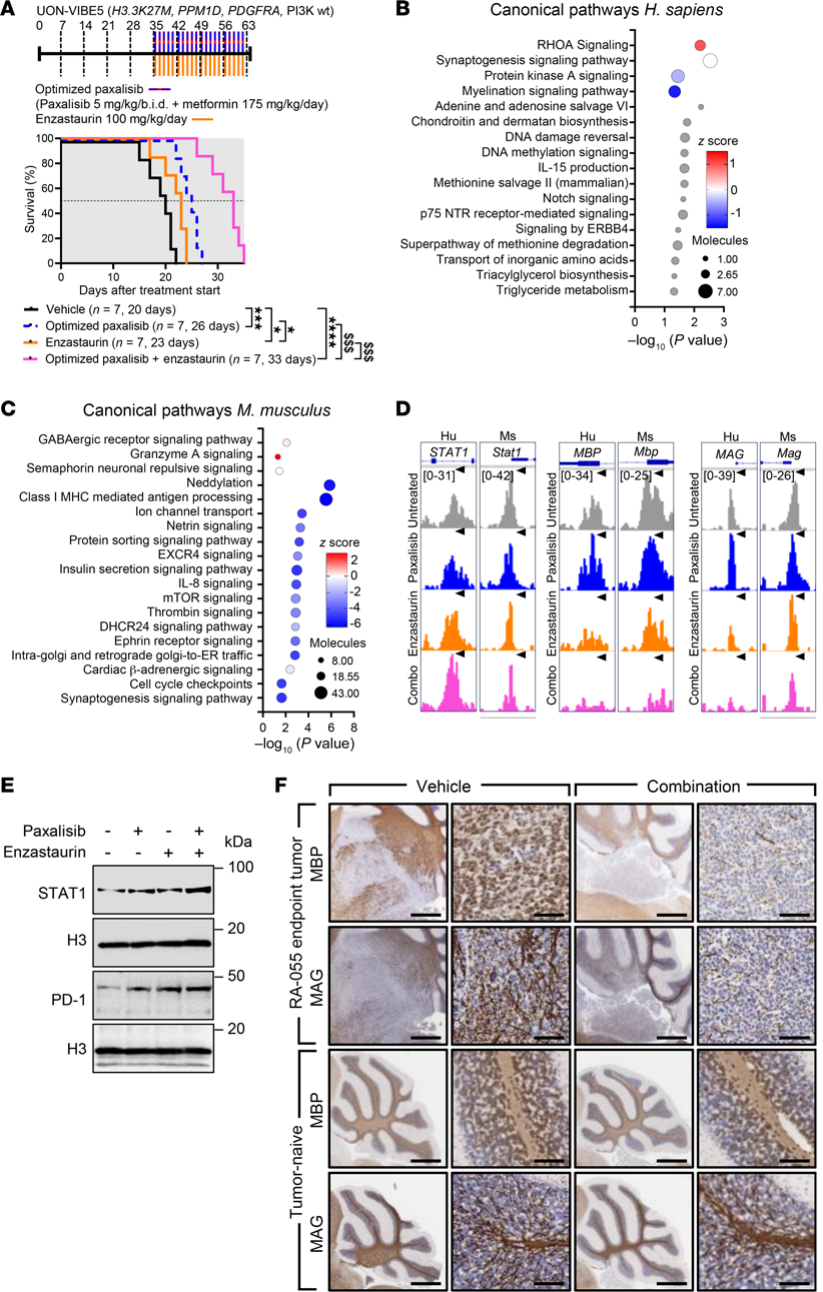
Assessment of open chromatin DIPG models treated at advanced disease stages identifies altered tumor myelination and interactions with the tumor immune microenvironment. (**A**) Kaplan-Meier survival analysis of UON-VIBE5 xenografts treated with the optimized combination of paxalisib (5 mg/kg/b.i.d.) + metformin (175 mg/kg/day), and enzastaurin (100 mg/kg/day) (shaded area indicates treatment time, log-rank test, treated versus untreated; *P < 0.05, **P < 0.01, ***P < 0.001,****P < 0.0001, synergistic comparisons; ^$$^*P* < 0.01, ^$$$^*P* < 0.001). IPA canonical pathway analysis of significantly altered pathways following combination treatment of (**B**) human and (**C**) mouse genes from peaks located at promoter and enhancer regions. (**D**) Representative tracks for selected genes of interest visualized by integrative genomics viewer, and corresponding (**E**) immunoblot validation. (**F**) IHC validation of MBP and MAG expression in RA-055-engrafted mice and tumor-naive C57BL/6J mice treated with the combination of optimized paxalisib and enzastaurin (*n* = 3 per treatment, representative images shown, scale bar: 50 μm).

Often, experimental therapies are first tested in clinical trials for DIPG patients at disease progression after RT. Therefore, to test if our optimized combination would provide a survival benefit to patients at disease progression/advanced disease, mice were xenografted with the patient-derived DIPG autopsy cell line (UON-VIBE5; H3.3K27M, *PDGRFA*, *PPM1D*), and treated with a continuous treatment regimen commencing at first sign of DIPG onset (Figure 7A). Vehicle treated mice succumbed 20 days after commencement of treatment. Optimized paxalisib and enzastaurin both provided a significant survival benefit as monotherapies (with medians of 26 days and 23 days, respectively). The combination synergistically extended survival compared with each monotherapy (median 33 days), highlighting the preclinical potential of this combination for the treatment of patients with DIPG in the upfront or advanced disease setting.

To assess how this regimen influenced chromatin accessibility and adaptive responses to our optimized regimen, we subjected UON-VIBE5 autopsy tissue to ATAC-Seq while on treatment and compared them to vehicle treated brainstems at endpoint (Figure 7, B–D). A modest decrease in chromatin accessibility was seen at enhancers in DIPG tissues treated with the combination (Supplemental Figure 9, A and B and Supplemental Table 9). ATAC-Seq identified 217 differentially expressed peaks (9% in promoters, *n* = 21 and 91% in enhancers, *n* = 194). However, this was more pronounced in the surrounding murine tumor microenvironment (TME) (Figure 7, C and D, Supplemental Figure 9, C and D, and Supplemental Table 10), with 1,703 differentially expressed peaks (43% in promoters, *n* = 738 and 67% in enhancers, *n* = 965). Canonical pathway analysis of differentially expressed peaks at human DIPG gene promoters and enhancers showed downregulation of key treatment targets, including PI3K and PKC, supporting therapeutic engagement (Figure 7B), while upregulated *Mus musculus* pathways of the TME were related to cytokine and antigen presentation (Figure 7C), specifically promoters and enhancers linked to NK signaling, NGF, AMPK and glutamatergic signaling pathways (Figure 7C and Supplemental Table 10).

In DIPG tissues, the combination was predicted to increase the activation of the Rho GTPases (RHOA) previously implicated in STAT1 activation (Figure 7, B, D, and E), contributing to changes in cellular morphology that lead to motility and invasion of glioblastoma cells (53, 54). Encouragingly, corresponding accessibility to promoters and enhancers of genes identified by scSTA was also seen via ATAC-Seq. These included *STAT1*, *MBP*, and *MAG* (Figure 7D), combining to promote increased expression of the immune checkpoint protein PD-1 (*PDCD1*; Figure 7E). As DIPG arises in cells of oligodendroglial linage (55, 56) that are critical for myelin development throughout childhood and adolescence (57), long-term treatment with the combination may promote DIPG cell demyelination (Figure 6, E and F and Figure 7D). To assess if demyelination occurred throughout the normal brain after treatment with the combination, we assessed MAG and MBP expression via IHC using both RA-055 xenograft tumors collected at endpoint and tumor naive C57BL/6J mice, treated with the combination for 4-weeks. Treatment decreased expression of MBP and MAG in RA-055 xenograft tissue (Figure 7F) in line with the UON-VIBE5 model (Figure 7, B and D), however, expression did not change in normal mouse brain tissues (Figure 7F), suggestive of a tumor-specific effect. Collectively, these data highlight the on-target treatment effects of our combination leading to adaptation through changes in tumor cell myelination and interactions with TME and highlight future potential consolidation treatment strategies.

### The addition of RT to the combination of paxalisib and enzastaurin is cytotoxic to DIPG in an immunocompetent setting.

**Figure 8:**
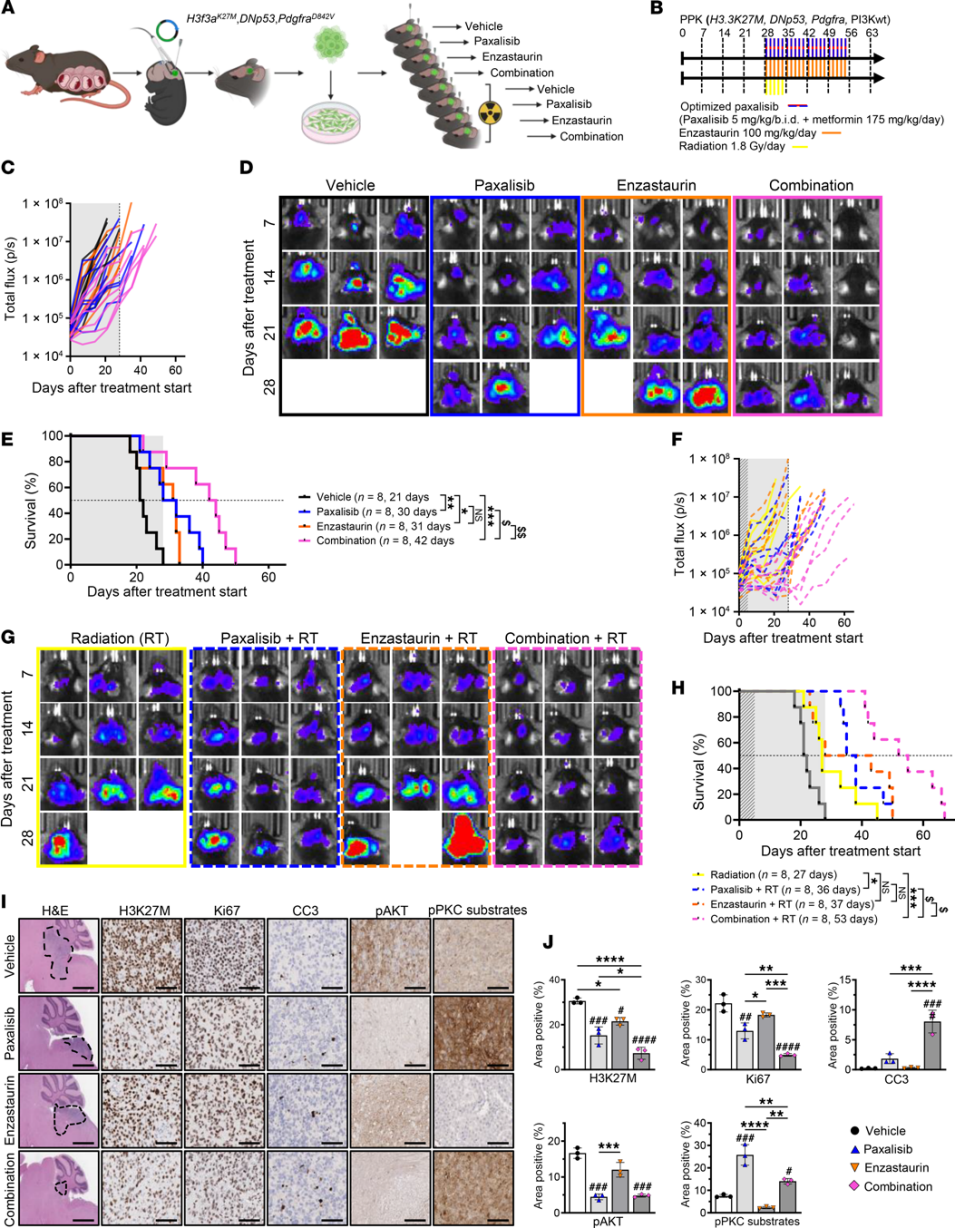
Combining paxalisib and enzastaurin with RT using an immunocompetent syngeneic DIPG mouse model. (**A**) In utero electroporation syngeneic allograft model of DIPG serially transplanted into C57BL/6J mice, (**B**) treated with optimized paxalisib (5 mg/kg/b.i.d. paxalisib + 175 mg/kg/day metformin) and enzastaurin (100mg/kg/day), alone and in combination with RT (1.8 Gy/day), for 4 weeks. (**C**) Monitoring of tumor burden using BLI over time (representative BLI images presented, shaded area indicates treatment time), (**D**) of mice treated with optimized paxalisib, enzastaurin, or the combination without RT. (**E**) Kaplan Meier survival analysis of mice treated with optimized paxalisib, enzastaurin, or the combination (shaded area indicates treatment time, log-rank test, treated versus untreated; *P < 0.05, ***P < 0.001, ****P < 0.0001, synergistic comparisons; ^$^*P* < 0.01, ^$$^*P* < 0.01). (**F**) Monitoring of tumor burden using BLI over time (representative images presented) (**G**) of mice treated with optimized paxalisib, enzastaurin ± RT. (**H**) Kaplan Meier survival analysis of mice treated with optimized paxalisib, enzastaurin, or the combination, with upfront RT (shaded area indicates treatment time, log-rank test, *P < 0.05, ***P < 0.001, ****P < 0.0001, synergistic comparisons; ^$^*P* < 0.01, ^$$^*P* < 0.01). Vehicle Kaplan-Meier curve is duplicated from (**E**) for visual reference. (**I**) IHC analysis of tumors resected 2 weeks after treatment and (**J**) quantified using ImageJ (measured in technical triplicate, across biological replicates, *n* = 3, 1-way ANOVA, *P < 0.05, **P < 0.01, ***P < 0.001, treated versus untreated; ^#^*P* < 0.05, ^##^*P* < 0.01, ^###^*P* < 0.001, ^####^*P* < 0.0001).

Using a syngeneic model of DIPG developed by in utero electroporation and serial transplantation of transduced cells, orthotopically engrafted into the brainstem of C57BL/6J mice, we assessed the benefit of RT alone and in combination with optimized paxalisib, enzastaurin, and the combination of both in an immunocompetent setting (Figure 8, A and B). Monitoring of tumor burden using bioluminescence imaging (BLI) showed a synergistic reduction in tumor burden using the combination of optimized paxalisib and enzastaurin compared with monotherapies (Figure 8, C and D). At treatment cessation, mice began to succumb to disease, with the combination of optimized paxalisib and enzastaurin significantly extending survival (42 days) compared with paxalisib and enzastaurin (30 and 31 days, respectively) alone, a doubling of survival compared with the vehicle-treated mice (21 days) (Figure 8E). The benefit of the combination was further potentiated using RT, driving tumor regression while on therapy (Figure 8, F and G), and significantly extending survival (53 days) compared with optimized paxalisib combined with RT (36 days), and enzastaurin combined with RT (37 days), doubling the survival advantage compared with RT alone (27 days), and trebling the survival benefit compared with the vehicle (Figure 8H). Analysis of tumors resected 2-weeks after treatment identified a significant reduction in tumor size and decreased tumor markers, including H3K27M and Ki67 (Figure 8, I and J). Encouragingly, our optimized regimen in combination with RT promoted in vivo apoptosis and led to a large reduction in tumor volumes (Figure 8, F–J).

### The combination of optimized paxalisib and enzastaurin is nontoxic in preclinical models.

Side effects induced by experimental treatment strategies impact the clinical benefit of potentially exciting approaches, therefore, we assessed the toxicity of the combination of optimized paxalisib and enzastaurin using immunocompetent C57BL/6J mice. Optimized paxalisib used in combination with enzastaurin for 4 weeks of continuous treatment showed no obvious systemic effects, including no change in red blood cell, platelet, hemoglobin, or thrombopoietin counts (Supplemental Figure 10, A–D). Our regimen did not alter normal organ morphology in the brain, liver, kidney, or spleen (Supplemental Figure 10E), highlighting the potential of these regimen to be tested in the clinic.

## Discussion

DIPG is an insidious disease responsible for more deaths in children than any other cancer (11, 25, 58). The loss of the heterochromatin mark H3K27me3 instigated by H3K27-alterations is the hallmark of DIPG, promoting euchromatin (5) and driving transcriptional programs that promote cellular immortality (59). Transcriptional volatility, coupled with cooccurring somatic mutations in tumor suppressor and signaling genes (3), offers some explanation as to why standard-of-care RT provides a transient benefit, and monotherapeutic treatment strategies have failed patients with DIPG (11, 25, 26). The PI3K/Akt/mTOR signaling cascade lies immediately downstream of many upregulated and mutant growth factor receptors responsible for the transmission of oncogenic signals that promote proliferation, angiogenesis, and metabolism, making this pathway an attractive therapeutic target. The importance of PI3K-mTOR signaling uncovered herein by analysis of loss-of-function CRISPR/Cas9 screen data, reveals both the catalytic p110α subunit of PI3K (*PIK3CA*) and downstream serine/threonine protein kinase *MTOR* are required to sustain DIPG cell growth and proliferation in vitro (Figure 1A and Supplemental Figure 1A). Interestingly, *PIK3CA* dependency was not mutation dependent and validated in patient-derived DIPG models (Figure 1, A–J and Supplemental Figure 1A). This was unlike data analyzed from the PRISM high-throughput genotype-specific cancer vulnerabilities database (60), showing that cancer cell lines harboring hotspot mutations in *PIK3CA* were more sensitive to paxalisib compared with cancers expressing WT-*PIK3CA* (94 versus 771, respectively), particularly in breast and ovarian cancers. However, analysis of diffuse gliomas included in this database showed no difference in paxalisib sensitivity between cell lines harboring mutant- and WT-*PIK3CA*, adding further relevance of targeting this pathway across patients with DIPG. Hence, we focused these studies on optimizing the brain penetrant PI3K-inhibitor paxalisib (18), which showed accumulation in the brainstem following the dose optimization regimen identified herein (Figure 2D).

Activated PI3K/Akt signaling, through PIK3CA and RAC-β serine/threonine-protein kinase (AKT2), mediates insulin-driven glucose uptake in muscle, liver, and fat cells, following translocation of glucose transporters to the plasma membrane (61). Hence, PI3K/Akt inhibition blocks insulin-driven glucose uptake, resulting in a dose-dependent increase in plasma levels of fasting C-peptide and insulin, thus causing hyperglycemia (62). Insulin is a systemic obligatory on-target pharmacodynamic surrogate for PI3K inhibition, activating the insulin receptor and reactivating PI3K/Akt signaling, particularly as DIPG is characterized by an abundance of insulin receptors (63, 64), potentially limiting the clinical benefit of PI3K antagonists (61).

Indeed, PI3K/Akt pathway inhibitors commonly cause toxicities that are dose limiting. Phase 1b clinical trials testing safety, tolerability, and PK, and to estimate the MTD of paxalisib administered immediately after RT in the pediatric DIPG setting (NCT03696355), identified DLTs including hyperglycemia and mucositis (19). This clinical trial established a safe dose of 27 mg/m^2^/day, equating to an equivalent mouse dose of approximately 9.2 mg/kg/day (30). Here, mice treated with 10 mg/kg/day showed significantly elevated blood glucose and C-peptide levels, indicative of hyperglycemia and hyperinsulinemia (Figure 2, I–L). By contrast, an optimized 5 mg/kg/b.i.d. dosing regimen decreased blood glucose levels below that recognized as hyperglycemic, but still elevated compared with vehicle control–treated mice. Such results raise the prospect of exploiting treatment paradigms that combine the use of antiglycemic approaches, such as metformin, in tandem with paxalisib to maintain glucose homeostasis, or a ketogenic diet in the adult brain cancer setting (NCT05183204).

The improved 5 mg/kg/b.i.d. regimen increased the survival of mice compared with the 10 mg/kg/day, suggestive of an accumulation of paxalisib in the brainstem (Figures 2D and Figure 3, A and B) and sustained inhibition of PI3K/Akt signaling (Figure 2, E and F and Figure 3, C and D). This benefit was potentiated using systemic control of insulin via metformin; however, this response was restricted to the use of either 5 mg/kg/day or 5 mg/kg/b.i.d. paxalisib. Metformin’s primary target is the liver, where it decreases hepatic gluconeogenesis and stimulates glucose uptake in muscle. Like the liver, treatment of DIPG orthotopic xenograft mouse models promoted AMPK independent phosphorylation and activation of the mTOR tumor suppressor TSC2 (65), thereby decreasing mTOR activity and protein synthesis, and providing additional control over DIPG cell growth and survival (Figure 3, C and D). Previous PK studies in the brain of mice showed metformin to be active in the CNS and to reach midline structures at a plasma to brain ratio of 1:1 (66).

Studies have shown in vitro anti-DIPG benefits from the combination of metformin and the pyruvate dehydrogenase kinase inhibitor dichloroacetate. This strategy decreased proliferation and promoted apoptosis; however, in this setting metformin did not significantly improve the survival of DIPG xenograft mice when used alone at 125 mg/kg/b.i.d. (67). Initially, we performed studies using the combination of paxalisib 5 mg/kg/b.i.d. and metformin 250 mg/kg/day, in line with studies that showed that this dose achieved potent mTOR antagonism in vivo (65). However, mice lost weight rapidly; therefore, we reduced the dose of metformin to 175 mg/kg/day, alone and in combination with paxalisib 5 mg/kg/b.i.d. This dose of metformin equates to approximately 14 mg/kg/day or 840 mg/60 kg adult, analogous to clinical dosing for patients with type 2 diabetes (68). The combination of metformin and paxalisib extended the survival of mice synergistically compared with both monotherapies (Figure 3, A and B), thus serving as an important consideration for ongoing clinical studies testing paxalisib.

DIPG displays a high degree of intratumoral clonal diversity (10, 25, 69), highlighting the necessity to develop effective combination strategies to improve survival. Focal gains in *PDGFRA*, *EGFR,* and *VEGFR* are seen in approximately 32% of DIPG cases, with PI3K alterations including constitutive activating mutations in *PIK3CA*, *PIK3R1,* and loss of function of *PTEN* (seen in 43% of patients combined), the latter associated with worse overall survival in DIPG (70). These PI3K alterations promote constitutive PI3K/Akt/mTOR signaling (11), pinpointing this signaling axis as a potential therapeutic strategy to improve outcomes. Among glioblastoma patients, 38% harbor an alteration in one or more PI3K pathway components, most commonly PTEN loss (approximately 30% of patients), followed by mutations in *PIK3CA* (13%), or *AKT1* (1%) (71). Brain development in the embryo is controlled partly by trophic factors (such as Insulin-like growth factor-1), hence neuronal cell survival is reliant on PI3K/Akt signaling (72). PI3K/Akt signaling is critical to the development of normal brain size and function during embryogenesis, highlighting the dependence of primitive neuronal stem cells on PI3K signaling for brainstem development, and the role PI3K plays in supporting the malignant growth of DIPG.

The novel application of RNA-Seq and phosphoproteomic profiling of DIPG cells treated with paxalisib identified increased Ca^2+^-activated PKC signaling. PRKCB (PKC-β) is a serine/threonine-protein kinase involved in various cellular processes, including insulin signaling, energy metabolism, and regulation of the B cell receptor (BCR) signalosome (73). PKC is also activated following the binding of brain-derived neurotrophic factor (BDNF) to neurotrophic receptor tyrosine kinase 2 (NTRK2, or TRKB), opening AMPAR channels to the postsynaptic membrane, again fundamentally regulated by Ca^2+^ (74). These processes are not only critical in the control of learning and behavior, but underpin neuron-DIPG and DIPG-DIPG communications (74). This encouraged us to test the CNS active PKC inhibitor enzastaurin in combination with paxalisib, which led to synergistic survival extension of DIPG xenograft models (Figure 5F, Figure 6A, Figure 7A, and Figure 8), highlighting the promise of what we believe to be a novel combination approach. Assessment of in vivo therapeutic adaption to our optimized combination showed decreased neuregulin signaling, suggesting, in part, that this approach may block known neuron-glioma communications.

Our in vitro data further showed the voltage-gated Ca^2+^ ion channel inhibitor gabapentin, combined synergistically with paxalisib, decreased the growth and proliferation of DIPG cell lines (Figure 4G). Future studies to inhibit the role that Ca^2+^ plays in the transmission of oncogenic signals in DIPG, in combination with optimized PI3K/Akt targeting, might therefore go some way toward improving response to these combinations. Hyperglycemia is a key factor responsible for the development of diabetic vascular complications through activation of PKC signaling (75). PKC propagates transmission of signals downstream of the insulin receptor, through a dose- and time-dependent increase in the phosphorylation of MARCKS (76) analogous to DIPG cells treated with paxalisib (Figure 4). Therefore, the use of antiglycemic therapies in combination with PI3K and PKC inhibitors may increase response. For example, metformin may further potentiate the therapeutic benefits of this multiagent, anti-DIPG strategy. Encouragingly, our biopsy-derived xenograft model treated with paxalisib and metformin in combination with the PKC-β inhibitor enzastaurin remained symptom free while on therapy (Figure 6A). This encouraged us to test the combination on our autopsy patient–derived xenograft model UON-VIBE5, established from a patient who received ONC201 as a monotherapy soon after the completion of RT and experienced stable disease for 24 months (42). We commenced treatment of this model with our optimized combination once mice showed signs of advanced disease (Figure 7A). Again, our optimized combination showed an encouraging survival benefit compared with each of the monotherapies, extending PDX survival by more 27%, and 65% compared with the vehicle control. In both PDX models treated with the combination, therapeutic plasticity was promoted by increased *STAT1* gene expression and an increased MHC II phenotype (Figure 6, E–G and Figure 7, D and E). IFN-γ directly promotes STAT1-mediated induction of immune effector genes, but whether this is mediated by cross regulation of DIPG responses by other cytokines and inflammatory factors secreted by cells of the TME is unknown.

Given that DIPG initiates in OPC-like cells, it was unsurprising that therapeutic adaptation modulated the myelin architecture of the tumor. PKC signaling in microglial cells promotes remyelination and repair of the CNS, therefore, systemic CNS PKC inhibition using enzastaurin may add to the complex milieu of secreted factors responsible for both therapeutic response and failure. Therefore, to achieve long-term survival it is an imperative to promote the benefit of RT, our only current weapon against DIPG. Using an immune competent syngeneic allograft DIPG mouse model, we subjected mice to our optimized combination with and without RT (Figure 8). Mice treated with the combination without RT experienced a doubling in survival, potentially promoted by the increased MHC II gene expression discovered using our PDX models (Figure 8E), while the inclusion of RT with the combination trebled the survival benefit, with most tumors showing impressive regression (Figure 8H). These results provide the impetus for future studies that may include the use of a STAT1 inhibitor, and/or checkpoint protein inhibitors either with the combination, as a maintenance strategy, or even in a metronomic manner, where repeated combinations are alternated to maintain long-term response. Nevertheless, these studies highlight the rational inclusion of clinically relevant therapies targeting the emerging biology revealed by our multiomic drug profiling strategy.

The preclinical investigation described herein has optimized paxalisib for the treatment of DIPG. Here, we have potentiated the on-target efficacy of the drug, while reducing common PI3K/Akt inhibitor related side effects and toxicities. Further, by performing multiomic analysis of DIPG models treated with paxalisib we have identified combination strategies that are targetable using FDA-approved therapies. Our data supports a model by which PI3K inhibition in DIPG drives calcium-dependent PKC activation, and in the process uncovers a clinically actionable combination strategy to inform future clinical trials.

## Methods

Detailed description of all materials and methods used throughout this study are in the Supplemental Materials.

### Sex as a biological variable.

Our study exclusively examined female mice, as all patient-derived DIPG cells used in this study were derived from female patients. It is unknown whether these findings are relevant in male mice.

### Statistics.

GraphPad Prism software (Version 9.1.0; La Jolla, CA, USA) was used to produce graphs and for statistical analysis of data. Unless otherwise stated, two sample, unpaired *t* tests or 1-way ANOVA were used to determine significant differences between groups. Event free survival analysis was performed using the Log-rank test. No data were excluded. Significant differences were detected in preliminary studies in our assays, prompting the use of minimum sample sizes for all in vivo experiments. In vitro experiments were performed at least 3 times each, per standard practices. Blinding was not performed in this study. Values shown are the mean ± SEM. *P* < 0.05 was considered significant.

### Study approval.

The use of patient-derived human DIPG/DMG cell lines was approved by the Human Ethics Research Committee, University of Newcastle (H-2018-0241), Human Ethics Research Committee, Monash University (HREC/17/MonH/323) and the Kantonale Ethikkommission Zurich (BASEC-Nr.2019-00615). All in vivo studies were approved by the University of Newcastle Animal Care and Ethics Committee (no. A-2019-900 and no. A-2020-004).

### Data availability.

Phosphoproteomics data is deposited to the ProteomeXchange via the PRIDE database (accession number PXD036114). Raw and processed BRB-Seq (accession number GSE211565) and ATAC-Seq files (accession no. GSE246057) are available for download from the Gene Expression Omnibus (GEO). All supporting values for this manuscript are provided in the Supporting Data Values file. Any other supporting data is available upon reasonable request from the corresponding author.

## Author contributions

MDD, RJD, JN, and JEC conceived and designed the study. RJD, ERJ, SGP, DK, IJF, AM, DES, BCT, ZPG, SL, PSK, MFBJ, AMD, TB, MLP, EAH, VJ, MA, CXS, PD, HPM, and DASB conducted the experiments. MDD, RJD, ERJ, SGP, RF, JEC, and JN interpreted the results. EEM, HCM, NMV, REV, BN, SH, CEB, YCS, FVM, MT, DSZ, EH, TNP, CVD, MJC, and SGG provided technical expertise. HER, NAV, CK, FA, CLT, HW, JRW, DDE, SM, SV, JRH, DMA and LBK provided clinical guidance. RJD, ERJ, and MDD designed the figures.

## Supplementary Material

Supplemental data

Unedited blot and gel images

Supplemental tables 1-3

Supporting data values

## Version 1 02/06/2024

In-Press Preview

## Version 2 03/15/2024

Electronic publication
