# Supplementary material for: PI3K/mTOR is a therapeutically targetable genetic dependency in diffuse intrinsic pontine glioma
Source: J Clin Invest. 2024 Feb 6;134(6):e170329. doi: 10.1172/JCI170329 (PMC10940093; doi:10.1172/JCI170329)
Supplement: Supplemental data [file jci-134-170329-s123.pdf]

Supplementary Information

Supplementary Tables

Supplementary Table ST1: Primary antibodies used for immunoblotting and immunohistochemistry.

Supplementary Table ST2: Cell line sensitivity to paxalisib.

Supplementary Table ST3: Xenium in situ spatial transcriptomic panel.

Supplementary Table ST4: Differential gene expression data for DIPG cells treated for 6 h with paxalisib.

Supplementary Table ST5: Differential gene expression data for DIPG cells treated for 12 h with paxalisib.

Supplementary Table ST6: Pooled differential gene expression data for DIPG cells treated for 6 and 12 h with paxalisib.

Supplementary Table ST7: Significantly regulated phosphorylation sites for DIPG cells treated for 6 h with paxalisib.

Supplementary Table ST8: Differential gene expression for Xenium in situ spatial transcriptomics.

Supplementary Table ST9: Differentially expressed human genes via ATAC-Seq.

Supplementary Table ST10: Differentially expressed murine genes via ATAC-Seq.

Supplementary Materials and Methods

Sex as a biological variable

Our study exclusively examined female mice as patient derived DIPG cells used in this study; SU-DIPG-XIIIP*, RA-055, and UON-VIBE5 were derived from female patients. It is unknown whether these findings are relevant for male mice.

DIPG genetic dependency data

The genetic dependency data were derived from the Victorian Paediatric Cancer Consortium's Childhood Cancer Model Atlas (CCMA) Data Portal that contains dependency data on 352 genes across 38 DMG cell line models (1).

Cas9 Cell Generation and sgRNA design for *PIK3CA* knockdown

Cells were seeded in a 6-well plate (200,000 cells per well) and incubated overnight. Cells were then replenished with fresh complete media containing 5 µg/mL polybrene (ThermoFisher Scientific, Waltham, MA, USA). A 250 µL aliquot of lentiviral cocktail containing either Lenti-Cas9-Blast plasmid (SU-DIPG-XIII; Addgene, Watertown, MA, USA) or Lenti-Cas9-2A-Blast (SU-DIPG-XXXVI; Addgene) was supplemented into the cell media and incubated for 72 h. Transduced cells were selectively maintained in complete media containing 10 µg/mL blasticidin (Jomar Life Research, Scoresby, Victoria, Australia) for at least 7 days. *PIK3CA* and non-targeting control (NTC) single guide RNA (sgRNA), cloned into the U6-gRNA/hPGK-puro-2A-BFP vector, were obtained from the Human Sanger Whole Genome Lentiviral CRISPR Library (ThermoFisher Scientific). The gRNA sequence for *PIK3CA* was 5'-GCAAATAATAGTGGTGATCTGG-3', and the non-targeting control (NTC) 5'-CCCAACTTCAACACCAATCT-3'. A total of 5×10^5 Lenti-X HEK29T were seeded in 6-well plates and the following day were transfected with sgRNA plasmids along with the viral packaging plasmids, psPAX-D64V (Addgene) and pMD2.G (Addgene) using Lipofectamine™ LTX Reagent with PLUS™ reagent as per the manufacturer's recommendations. Transfection media was replaced with fresh media after 6 h and incubated for a further 72 h prior to collection of virus-containing media. Viral media was added to 2×10^5 Cas9-expressing DIPG

cells in a 6-well plate in the presence of 1 µg/mL polybrene, centrifuged at 800 x g for 30 min and then incubated for 72 h. Selection of transduced cells using 2 µg/mL of puromycin in fresh media was performed until non-transduced control cells were dead. Heterogenous cell lines were maintained in 2 µg/mL puromycin. For the establishment of single cell clones from the heterogenous population, single blue fluorescent protein (BFP) positive cells were sorted in 96-well plates containing a 1:1 mixture of conditioned media and fresh media. Single cell clones were expanded and screened using immunoblotting to identify clones with reduced or absent PIK3CA protein.

High-throughput drug screening

Cellular growth and proliferation of DIPG neurosphere cell lines was determined using a resazurin growth and proliferation assay, as previously described (2).

TSO500 next generation sequencing analysis

Genomic profiling was performed on 16 DMG neurosphere cell lines using an Illumina TruSight Oncology 500 (TSO500, Illumina, CA, USA) next-generation sequencing (NGS) assay as previously described (3). Following FASTQ validation, sequences were aligned to the hg19 genome (4). PISCES was used to perform somatic variant calling to identify variants at low frequency, which are filtered when error rates do not meet quality thresholds (5). GEMINI was used to perform local INDEL realignment, paired-read stitching, and read filtering to identify insertion and deletion events as well as further improve variant calling results (6). The CRAFT copy number (CN) variant caller performed amplification, reference, and deletion calling for target copy number variation (CNV) genes within the assay (3). The CRAFT software component counts the coverage of each target interval on the panel, performs normalization, calculates fold change values for each gene, a quality control Q-score and determines the CNV status for each CNV target gene. Illumina Annotation Engine Nirvana software performed annotation of small variants, calculating tumor mutational burden and microsatellite instability for each sample (7). Finally, the MANTA fusion caller was utilized to discover, assemble, and score large-scale gene fusions (8). TSO500 contamination detection was implemented to

identify contaminated samples by examining a combination of contamination p-value (p-score) and scores determined by error rate per sample and total read depth of the nucleotide position. VCF files for each sample produced by TSO500 were analyzed using the Helium application, allocating a Helium Score (a measure of likely pathogenicity of the variant based on its appearance in somatic mutation databases: COSMIC, Clinvar, CGC and CADD), and Global Max Allele Frequency (frequency of that variant within the global population), to each variant (9). Variants with a Global Max Allele Frequency of $<1e^{-5}$ were predicted to be somatic unless present in a matched germline sample. CNVs for each gene were grouped into categorical states (copy number deletion [CN<1.32], copy number loss [CN between 1.32 and 1.7], no change [CN between 1.7 and 3], copy number gain [CN between 3 and 7] and copy number amplification [CN>7]) based on categories from genomic studies (10, 11). Predicted somatic variants and CNVs with a Q-score >125 in genes belonging to the PI3K/AKT/mTOR signaling pathway were plotted in an Oncoprint using the ComplexHeatmaps package (12).

Immunoblotting

Protein was extracted from DIPG cells using RIPA buffer and previously described (13). Protein quantification was performed using a Pierce BCA Protein Assay Kit (ThermoFisher Scientific) according to the manufacturer's instructions. For sample gel loading, 15 µg of protein was denatured using final 1x concentration of LDS loading sample buffer containing 8% of β-mercaptoethanol, by boiling at 100°C for 5 minutes. Proteins were separated using 1x MOPS running buffer (200 mM MOPS, 50 mM Sodium acetate, 10 mM EDTA at pH 7.0 makes 10x MOPS) via electrophoresis using NuPAGE NOVEX Bis-Tris 4% to 12% gels (Invitrogen) in accordance with the methods described by Laemmli (14). Proteins were transferred to a nitrocellulose membrane with transfer buffer (25 mM Tris, 192 mM glycine, pH 8.3 with 20% methanol [vol/vol]), using standard immunoblotting transfer techniques. Nitrocellulose blots were blocked with 5% skim milk in TBS-T at room temperature for 1 h. Primary antibodies were incubated overnight at 4 °C at dilutions described in Supplementary Table ST1. Secondary horseradish peroxidase (HRP) conjugated antibody (Bio-Rad,

Hercules, CA, USA) was used at a dilution of 1:5000 (antibodies in Supplementary Table ST1). Protein cross-reactivity with selected antibodies was detected using enhanced chemiluminescence reagents (Merck, Burlington, MA, USA) prior to imaging with a Chemidoc MP Imaging System (Bio-Rad) and densitometry analysis with ImageLab software (Bio-Rad).

Bulk RNA barcode (BRB) sequencing

BRB-sequencing (henceforth referred to as RNA-seq) was performed as previously described (15). The SU-DIPG-VI neurosphere cell line was seeded in 6-well plates (500,000 cells per well) and treated with paxalisib at IC_{50} (Supplementary Table ST2). Cells were collected 0, 6 and 12 h post treatment. RNA-seq libraries were prepared with a Mercurius BRB-seq kit for 96 samples (Alithea Genomics, Epalinges, Switzerland) (16). RNA library quality was assessed using Agilent TapeStation (Agilent, Santa Clara, CA, USA). Fragments between 300–1000 bp were included and quantified using a Qubit High Sensitivity Assay (ThermoFisher Scientific). Pooled samples were sequenced to a depth of 441.4 million reads using the NextSeq v2.5 High Output (75 cycles) on the Illumina NextSeq 500 (Illumina, San Diego, CA, USA). Volcano plots of the differential expression analysis of each treatment relative to DMSO control were constructed, and the threshold of significance for each gene was set to a false-discovery rate adjusted p-value (FDR) of less than 1 and an absolute $\log_2$ fold change greater than 1 (17). For heatmap visualization and clustering, datasets of each treatment along with control were normalized using variance stabilizing transformation from DESeq2, and batch effects were removed using the remove batch effect function in limma (18). Differentially expressed genes were analyzed using Ingenuity Pathway Analysis (IPA) software.

Orthotopic DIPG syngeneic allograft and patient derived xenograft (PDX) mouse models

A single cell suspension of SU-DIPG-XIII-P* (500,000 cells), HSJD-DIPG-007 (200,000 cells), RA-055 (200,000 cells), or UON-VIBE5 (300,000 cells) patient derived neurosphere cell lines were injected into the pontine region of 6–8-week-old, female, NSG (NOD-*scid* IL2Rgamma^{null})

mice under isoflurane anesthesia. IUE-24 cells (hereby referred to as PPK) were generated by intra-uterine electroporation of *H3f3a*^{K27M}, *Pdgfra*^{D842Vmut}, *Trp53*$^{-/-}$, into the brainstem of C57BL/6J neonates at embryonic day 13.5 as previously described (19), *ex vivo* cultured and implanted (300,000 cells) into the pontine region of 6-8 week old, female, C57BL/6J mice under isoflurane anesthesia. Stereotactic coordinates were 0.8 mm to the right of midline, 0.5 mm posterior to lambda and 5 mm dorsal to ventral. For all immunocompromised PDX studies, animals were randomly allocated to a treatment group. For the immunocompetent PPK model mice were imaged by bioluminescence on day 28 and randomized into treatment groups based on average signal before treatment commencement. Mice were treated with paxalisib (5 mg/kg/day, 5 mg/kg/b.i.d. or 10 mg/kg/day as specified), metformin (150 mg/kg/day), enzastaurin (100 mg/kg/day), ribociclib (75 mg/kg/day) or vandetanib (25 mg/kg/q.w.), or a combination as specified, by oral gavage in 1% methylcellulose cp15/0.2% Tween 80 (5 days on, 2 days off) continuously or for a maximum of five weeks. Whole brain radiation was performed using a MultiRad225 (Precision X-Ray, Madison, CT, USA) and 1.8 Gy was delivered daily for 5 days using body shields. If required, mice were given dose holidays after 10% weight loss on therapy, recommencing after 5% of weight was regained. Mice were euthanized at an ethical end point, including at signs of neurologic decline, such as ataxia, circling or head tilting, with or without 20% weight loss. Overall survival was determined via the Kaplan-Meier survival analysis. GraphPad Prism Version 9.1.0. was used for in vivo statistical analyzes using the log-rank (Mantel-Cox) test. In all cases, values of $p<0.05$ were regarded as being statistically significant compared to vehicle, and $p<0.01$ considered the threshold for combination synergism compared to monotherapies as described in (20).

Pharmacokinetic (PK) and Pharmacodynamic (PD) analysis

For pharmacokinetic (PK) studies, paxalisib was administered as a single dose via oral gavage to 6-8-week-old, female, NSG mice at either 5 mg/kg, 10 mg/kg or two 5 mg/kg doses 12 h apart (5 mg/kg/b.i.d.). After 1, 6 and 24 h post treatment, mice were sacrificed by CO_2 euthanasia. Immediately following, blood was extracted via cardiac puncture, and brains

collected. Blood plasma was separated via centrifugation (2,500 × *g*, 4°C, 15 min) and frozen at -80°C. Brainstem, thalamus, and prefrontal brain regions were dissected prior to snap freezing in liquid nitrogen. Brain tissues were homogenized using Lysing Matrix beads in a FastPrep-24™ 5G system (MP Biomedicals, Irvine, CA, USA) at the factory recommended settings. Paxalisib was purified from plasma and brain homogenates using a protein precipitating mixture composed of 90% v/v acetonitrile, 10% v/v ethanol and 0.1% v/v glacial acetic acid. The supernatant was separated and collected following centrifugation. The supernatants were analyzed using a Nexera X2 UHPLC system (Shimadzu, Kyoto, Japan) coupled to a QTRAP 6500 System (SCIEX, Framingham, MA, USA) via multiple reaction monitoring (MRM) using 282.0 and 240.1 transitions of the 367.2 m/z precursor mass as described (21). Quantitative analysis was conducted using MultiQuant Software (SCIEX) against a calibration curve for paxalisib over the concentration range of 0.061 – 1000.0 pg/µL (21).

To assess the pharmacodynamics (PD) properties of paxalisib, NSG mice were engrafted with the SU-DIPG-XIII-P* neurosphere cell line as described. Two weeks post xenograft, mice were treated with a single dose of paxalisib at 5 mg/kg, 10 mg/kg or two 5 mg/kg doses 12 h apart (5 mg/kg/b.i.d.), by oral gavage, before being sacrificed by CO_2 euthanasia, 6, 12, or 24 h later. Immediately following euthanasia, mice were transcranial perfused with saline, brains removed, brainstem and prefrontal cortex dissected and snap frozen in liquid nitrogen. Tissue was homogenized on ice using a Dounce homogenizer in RIPA protein extraction buffer containing protease and phosphatase inhibitors. Homogenized samples were sonicated 5 × 20 sec at 4°C, before being clarified through centrifugation at 25,000 × *g* for 30 min at 4°C. Samples were then subjected to standard immunoblotting techniques as described above (13) using appropriate combinations of primary and secondary antibodies (Supplementary Table ST1).

Blood glucose and C-peptide analysis

NSG or C57BL/6 mice were treated with paxalisib at 5 mg/kg, 10 mg/kg or two 5 mg/kg doses 12 h apart (5 mg/kg/b.i.d.), by oral gavage, for two weeks (5 days on, 2 days off) before being sacrificed by CO_2 euthanasia, 6, 12, or 24 h post last dose. Blood was collected via cardiac puncture for measurement of blood glucose and C-peptide. Blood was loaded onto Freestyle Optium glucose test strips (Abbott, Abbott Park, IL, USA) and levels of blood glucose were measured using a Freestyle Optium Neo (Abbott). Blood was centrifuged (2,500 × *g*, 15 min, 4°C) and plasma supernatant was stored at -80°C. Mouse C-peptide ELISA kit (ALPCO, Salem, NH, USA) was used to quantify C-peptide levels in the plasma, as per manufacturer's recommendations.

Toxicity testing

Blood was collected via cardiac puncture and complete blood cell counts were performed using a Mindray BC-5000 auto hematology analyzer (MindRay, Shenzhen, P.R. China). Liver function tests and biochemistry profiling was conducted using a Vetscan VS2 biochemistry analyzer (Zoetis, Parsippany, NJ, USA) using a comprehensive profile kit, as per manufacturers recommendations. Mouse thrombopoietin was assessed via ELISA (Abcam). Differences in blood counts and liver biochemistry was compared to vehicle treatment mice. To assess normal organ function, organs were collected and prepared for staining with hematoxylin and eosin by the HMRI Core Histology facility with histology assessed by an anatomical pathologist from the Hunter Area Pathology service.

Immunohistochemistry

Immunohistochemistry optimization and staining services were provided by The University of Newcastle's 'NSW Regional Biospecimen Services'. Staining was performed on the Discovery Ultra Benchmark Immunohistochemistry Automated Platform (Roche). Briefly, antigen retrieval was conducted at 95°C for 32 mins, blocked at 37°C for 12 mins before addition of primary antibodies per Supplementary Table ST1. Anti-rabbit HQ was applied for 16 mins at 37°C followed by tertiary of HRP HQ for 16 mins at 37°C and developed using diaminobenzidine (DAB). Slides were counterstained using freshly filtered hematoxylin for 10

seconds, before being washed and dehydrated per standard procedures (Supplementary Table ST1).

High-resolution quantitative phosphoproteomic profiling

SU-DIPG-XXXVI cells were treated with paxalisib and subjected to proteomic and phosphoproteomic analysis as previously described (22-24). The SU-DIPG-XXXVI neurosphere cell line was treated with paxalisib at IC_{50} (Supplementary Table ST2) for 6 h. Tryptic peptides from each sample were prepared as described (23, 25, 26) individually labeled using tandem mass tags (TMT) and phosphopeptides were isolated from the proteome using titanium dioxide (25) and immobilized metal affinity chromatography before offline hydrophilic interaction liquid chromatography (HILIC) (2, 23, 24). Liquid chromatography (LC) tandem mass spectrometry (MS/MS) was performed using a Q-Exactive Plus hybrid quadrupole-Orbitrap MS system, coupled to a Dionex Ultimate 3000RSLC nanoflow HPLC system as described (23, 24). Protein abundances were generated from MS data using Proteome Discoverer 2.5 (ThermoFisher Scientific), filtered based on a false discovery rate (FDR) of 1% and normalized to spiked-in heavy labelled peptides. Differentially regulated pathways post treatment with paxalisib were identified using IPA software. Integrative Inferred Kinase Activity (INKA) (27) and PhoxTrack kinase activation analysis (28) were used to identify altered kinases as a result of treatment with paxalisib as previously described. (2)

Xenium in situ spatial transcriptomics

Brains from RA-055 tumor bearing xenografts were collected after four weeks of treatment with the combination of optimized paxalisib (5 mg/kg/b.i.d. with 150 mg/kg/day metformin) and enzastaurin (100 mg/kg/day) or once mice had reached endpoint, formalin fixed paraffin embedded (FFPE) and subjected to Xenium in situ spatial transcriptomics (10x Genomics, Pleasanton, CA, USA) as previously described. (29, 30) Briefly, initial data was generated by the Xenium Analyzer using 5 µM FFPE sections mounted to Xenium slides followed by deparaffinization and permeabilization to access mRNA. mRNA was targeted through a custom 358 gene panel (Supplementary Table ST3). Probes were hybridized at 50°C

overnight at a concentration of 10 nM, before washing and ligation at 37ºC for 2 h. Circularizable DNA probes were hybridized with target RNA and a gene-specific barcode. Two ends of the probes bound to the target RNA and ligated to generate a circular DNA probe. Following ligation, the circularized probe was amplified, producing multiple copies of the gene-specific barcode for each target mRNA. Fluorescently labeled oligos were bound to the amplified DNA probes. Cyclical rounds of fluorescent probe hybridization, imaging, and removal were used to generate optical patterns specific for each barcode. Cell segmentation was performed by staining DNA using DAPI (4',6-diamidino-2-phenylindole) to detect nuclei. Xenium output files were imported with R (4.3.1) using the LoadXenium function from Seurat (4.9.9.9050) (31). Cells were then filtered using a threshold of 10 UMI counts (absolute number of observed transcripts) minimum per cell. The imported cells were selected using the lasso tool in Xenium Explorer to highlight specific regions and clusters of interest, and cell identification was exported into .csv files and analyzed using Seurat. Differential gene expression (DGE) analysis between selected conditions was assessed using a Wilcoxon test on SCTransform normalized count data using the FindMarkers Seurat function (31). Violin plots were generated for all genes in the Xenium panel between conditions of interest.

Assay for transposase-accessible chromatin with sequencing (ATAC-seq)

Brainstems were collected from UON-VIBE5 xenograft bearing mice and flash frozen in liquid nitrogen, before being sent to Active Motif for ATAC-seq (Active Motif, Carlsbad, CA, USA). Briefly, tissues were manually disassociated, isolated nuclei were quantified using a hemocytometer, and 100,000 nuclei were tagmented as previously described (32), with some modifications based on Corces, et al. (33) using the enzyme and buffer provided in the ATAC-seq Kit (Active Motif). Tagmented DNA was then purified using the MinElute PCR purification kit (Qiagen, Hilden, Germany), amplified with 10 cycles of PCR, and purified using Agencourt AMPure SPRI beads (Beckman Coulter, Brea, CA, USA). Resulting material was quantified by Nanodrop and sequenced with PE42 sequencing on the NovaSeq 6000 sequencer (Illumina). Reads were aligned using the BWA (Burrows-Wheeler Aligner) algorithm (mem mode; default settings) to hg38 and mm10 reference genomes. Duplicate reads were

removed, and only reads mapping as matched pairs and only uniquely mapped reads (mapping quality ≥1) were used for further analysis. To generate bigwig coverage files, alignments were extended *in silico* at their 3'-ends to a length of 200 bp and assigned to 32-nt bins along the genome. The bigwig files were used to generate heatmaps and profile plots using the deepTools package and visualized using the Integrative Genome Viewer (IGV). Peaks were identified using the MACS 2.1.0 algorithm at a cutoff of p-value 1e-7, without control file, and with the nomodel option. Peaks that overlapped with the ENCODE blacklist were removed. Signal maps and peak locations were used as input data to Active Motifs proprietary analysis program to perform differential analysis. Differential regions between the untreated sample and the combination treatment were further filtered based on log fold change (>1.5 or <-1.5), peak length >100bp, and peak signal, before using their assigned genes for gene set enrichment analysis of canonical pathways.

Statistical analysis

GraphPad Prism software (Version 9.1.0; La Jolla, CA, USA) was used to produce graphs and for statistical analysis of data. Unless otherwise stated, two sample, unpaired t-tests or one-way ANOVA were used to determine significant differences between groups. Event free survival analysis was performed using the Log-rank test. No data were excluded. Significant differences were detected in preliminary studies in our assays, prompting the use of minimum sample sizes for all in vivo experiments. In vitro experiments were performed at least 3 times each, per standard practices. Blinding was not performed in this study. Values shown are the mean ± SEM. Significance values: * = $p<0.05$, ** = $p<0.01$, *** = $p<0.001$, **** = $p<0.0001$ are used throughout.

References

1. Sun C, et al. Generation and Multi-Dimensional Profiling of a Childhood Cancer Cell Line Atlas Defines New Therapeutic Opportunities. *Cancer Cell.* 2023.
2. Murray HC, et al. Quantitative phosphoproteomics uncovers synergy between DNA-PK and FLT3 inhibitors in acute myeloid leukaemia. *Leukemia.* 2020.
3. Pestinger V, et al. Use of an Integrated Pan-Cancer Oncology Enrichment Next-Generation Sequencing Assay to Measure Tumour Mutational Burden and Detect Clinically Actionable Variants. *Molecular Diagnosis & Therapy.* 2020;24(3):339-49.
4. Pan B, et al. Similarities and differences between variants called with human reference genome HG19 or HG38. *BMC Bioinformatics.* 2019;20(2):101.
5. Dunn T, et al. Pisces: an accurate and versatile variant caller for somatic and germline next-generation sequencing data. *Bioinformatics.* 2019;35(9):1579-81.
6. Paila U, et al. GEMINI: integrative exploration of genetic variation and genome annotations. *PLoS Comput Biol.* 2013;9(7):e1003153.
7. Stodolna A, et al. Clinical-grade whole-genome sequencing and 3' transcriptome analysis of colorectal cancer patients. *Genome Med.* 2021;13(1):33.
8. Chen X, et al. Manta: rapid detection of structural variants and indels for germline and cancer sequencing applications. *Bioinformatics.* 2016;32(8):1220-2.
9. McLaren W, et al. The Ensembl Variant Effect Predictor. *Genome Biol.* 2016;17(1):122.
10. Magi A, et al. Detecting common copy number variants in high-throughput sequencing data by using JointSLM algorithm. *Nucleic Acids Res.* 2011;39(10):e65.
11. Mackay A, et al. Integrated Molecular Meta-Analysis of 1,000 Pediatric High-Grade and Diffuse Intrinsic Pontine Glioma. *Cancer Cell.* 2017;32(4):520-37 e5.
12. Gu Z, et al. Complex heatmaps reveal patterns and correlations in multidimensional genomic data. *Bioinformatics.* 2016;32(18):2847-9.
13. Dun MD, et al. Investigation of the expression and functional significance of the novel mouse sperm protein, a disintegrin and metalloprotease with thrombospondin type 1 motifs number 10 (ADAMTS10). *Int J Androl.* 2012;35(4):572-89.
14. Laemmli UK. Cleavage of structural proteins during the assembly of the head of bacteriophage T4. *Nature.* 1970;227(5259):680-5.
15. Przystal JM, et al. Imipridones affect tumor bioenergetics and promote cell lineage differentiation in diffuse midline gliomas. *Neuro Oncol.* 2022;24(9):1438-51.
16. Alpern D, et al. BRB-seq: ultra-affordable high-throughput transcriptomics enabled by bulk RNA barcoding and sequencing. *Genome Biol.* 2019;20(1):71.
17. Schrode N, et al. Analysis framework and experimental design for evaluating synergy-driving gene expression. *Nat Protoc.* 2021;16(2):812-40.
18. Love MI, et al. Moderated estimation of fold change and dispersion for RNA-seq data with DESeq2. *Genome Biol.* 2014;15(12):550.
19. du Chatinier A, et al. Generation of immunocompetent syngeneic allograft mouse models for pediatric diffuse midline glioma. *Neurooncol Adv.* 2022;4(1):vdac079.
20. Rose WC, and Wild R. Therapeutic synergy of oral taxane BMS-275183 and cetuximab versus human tumor xenografts. *Clin Cancer Res.* 2004;10(21):7413-7.
21. Duchatel RJ, et al. Preclinical and clinical evaluation of German-sourced ONC201 for the treatment of H3K27M-mutant diffuse intrinsic pontine glioma. *Neurooncol Adv.* 2021;3(1):vdab169.
22. Dun MD, et al. Proteotranscriptomic Profiling of 231-BR Breast Cancer Cells: Identification of Potential Biomarkers and Therapeutic Targets for Brain Metastasis. *Mol Cell Proteomics.* 2015;14(9):2316-30.
23. Degryse S, et al. Mutant JAK3 phosphoproteomic profiling predicts synergism between JAK3 inhibitors and MEK/BCL2 inhibitors for the treatment of T-cell acute lymphoblastic leukemia. *Leukemia.* 2018;32(3):788-800.

24. Nixon B, et al. Modification of Crocodile Spermatozoa Refutes the Tenet That Post-testicular Sperm Maturation Is Restricted To Mammals. *Mol Cell Proteomics.* 2019;18(Suppl 1):S58-S76.
25. Staudt DE, et al. Phospho-heavy-labeled-spiketide FAIMS stepped-CV DDA (pHASED) provides real-time phosphoproteomics data to aid in cancer drug selection. *Clin Proteomics.* 2022;19(1):48.
26. Murray HC, et al. Synergistic targeting of DNA-PK and KIT signaling pathways in KIT mutant acute myeloid leukemia. *Mol Cell Proteomics.* 2023:100503.
27. Beekhof R, et al. INKA, an integrative data analysis pipeline for phosphoproteomic inference of active kinases. *Mol Syst Biol.* 2019;15(5):e8981.
28. Weidner C, et al. PHOXTRACK-a tool for interpreting comprehensive datasets of post-translational modifications of proteins. *Bioinformatics.* 2014;30(23):3410-1.
29. Mangoli A, et al. Ataxia-telangiectasia mutated (Atm) disruption sensitizes spatially-directed H3.3K27M/TP53 diffuse midline gliomas to radiation therapy. *bioRxiv.* 2023:2023.10.18.562892.
30. Janesick A, et al. High resolution mapping of the breast cancer tumor microenvironment using integrated single cell, spatial and in situ analysis of FFPE tissue. *bioRxiv.* 2022:2022.10.06.510405.
31. Hao Y, et al. Integrated analysis of multimodal single-cell data. *Cell.* 2021;184(13):3573-87.e29.
32. Buenrostro JD, et al. Transposition of native chromatin for fast and sensitive epigenomic profiling of open chromatin, DNA-binding proteins and nucleosome position. *Nat Methods.* 2013;10(12):1213-8.
33. Corces MR, et al. An improved ATAC-seq protocol reduces background and enables interrogation of frozen tissues. *Nat Methods.* 2017;14(10):959-62.

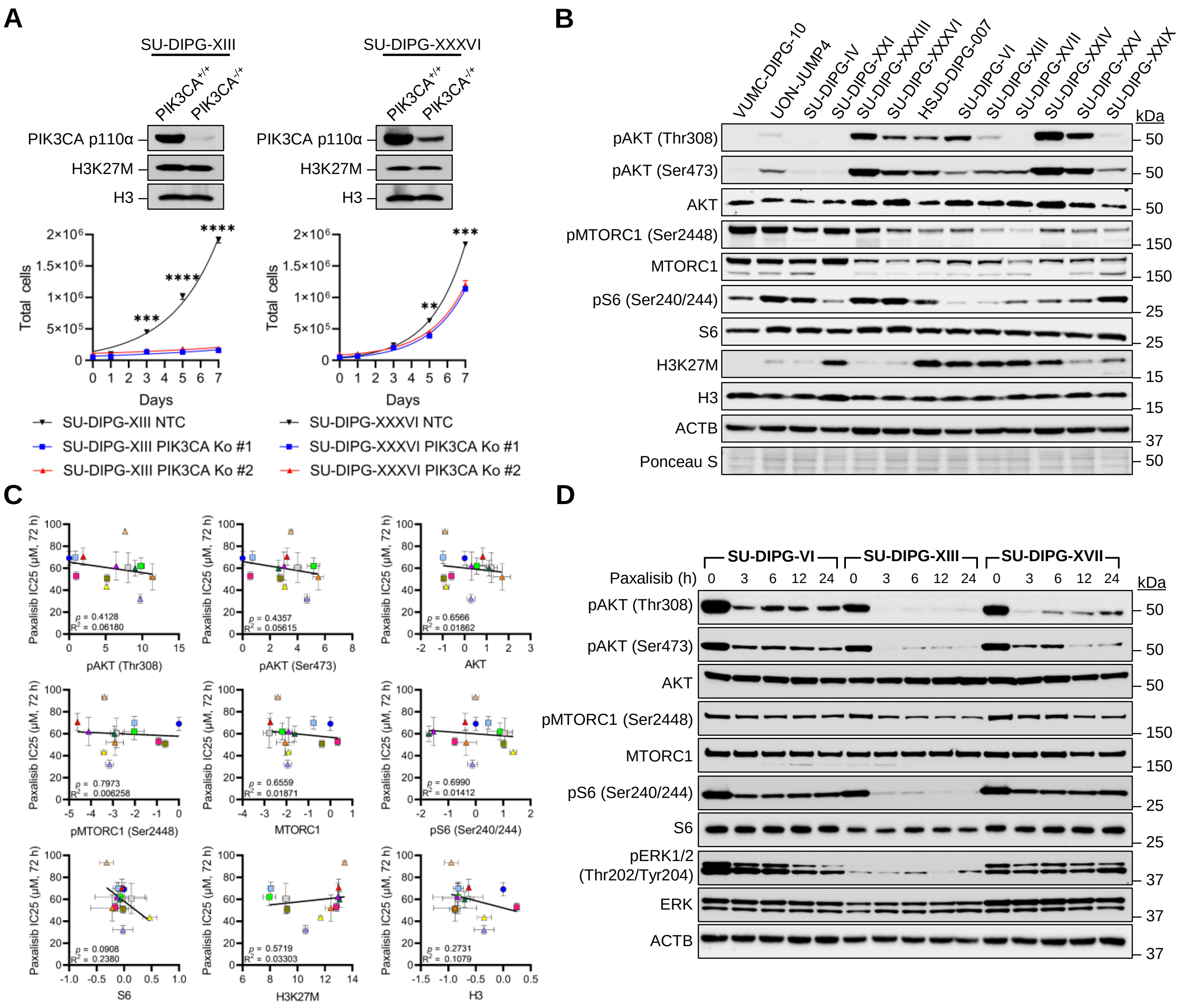

Supplementary Figure S1: Expression and phosphorylation of PI3K/Akt/mTOR signaling proteins does not predict sensitivity to with paxalisib in vitro. (A) Growth rates of SU-DIPG-XXXVI and SU-DIPG-XIII cells harboring *PIK3CA* knockout. (B) Protein expression of key PI3K/Akt/mTOR signaling proteins in DIPG cell lines measured by immunoblotting (n=3, representative immunoblots presented). (C) Densitometry of immunoblot values normalized to VUMC10 and correlated to IC25 values for paxalisib (significance determined using Pearson’s linear regression). (D) Protein phosphorylation of key PI3K/Akt/mTOR signaling proteins in DIPG cell lines (SU-DIPG-VI, SU-DIPG-XIII, SU-DIPG-XVII) treated with paxalisib over time (1 µM, n=3, representative immunoblots presented).

A

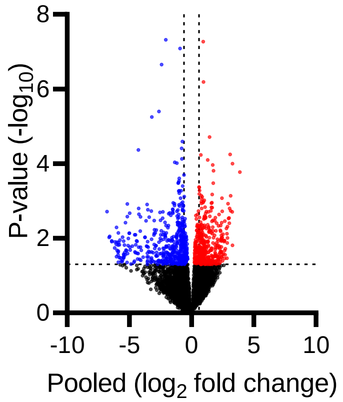

Total genes identified	12285
Total up-regulated	454 (3.7%)
Total down-regulated	526 (4.2%)

B

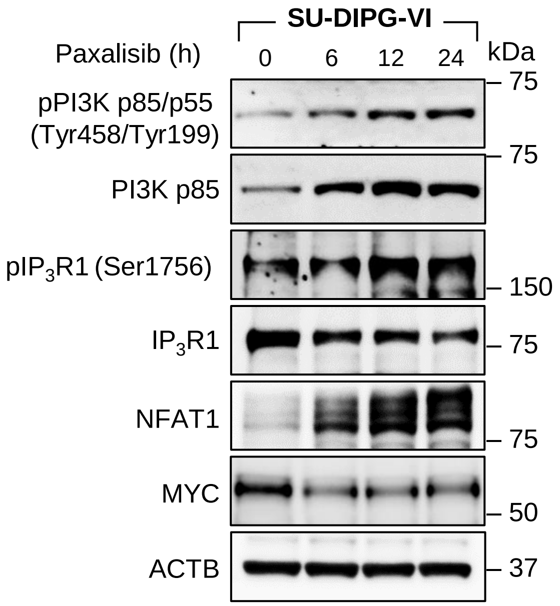

Supplementary Figure S2: Volcano plot analysis of transcription regulation following paxalisib treatment. (A) Bulk RNA Barcoding and sequencing (BRB-seq) of SU-DIPG-VI following paxalisib treatment (1 µM) for pooled data. (B) Validation of important oncogenes shown to be significantly modulated by paxalisib treatment at the protein level by immunoblotting (n=3, representative immunoblots presented).

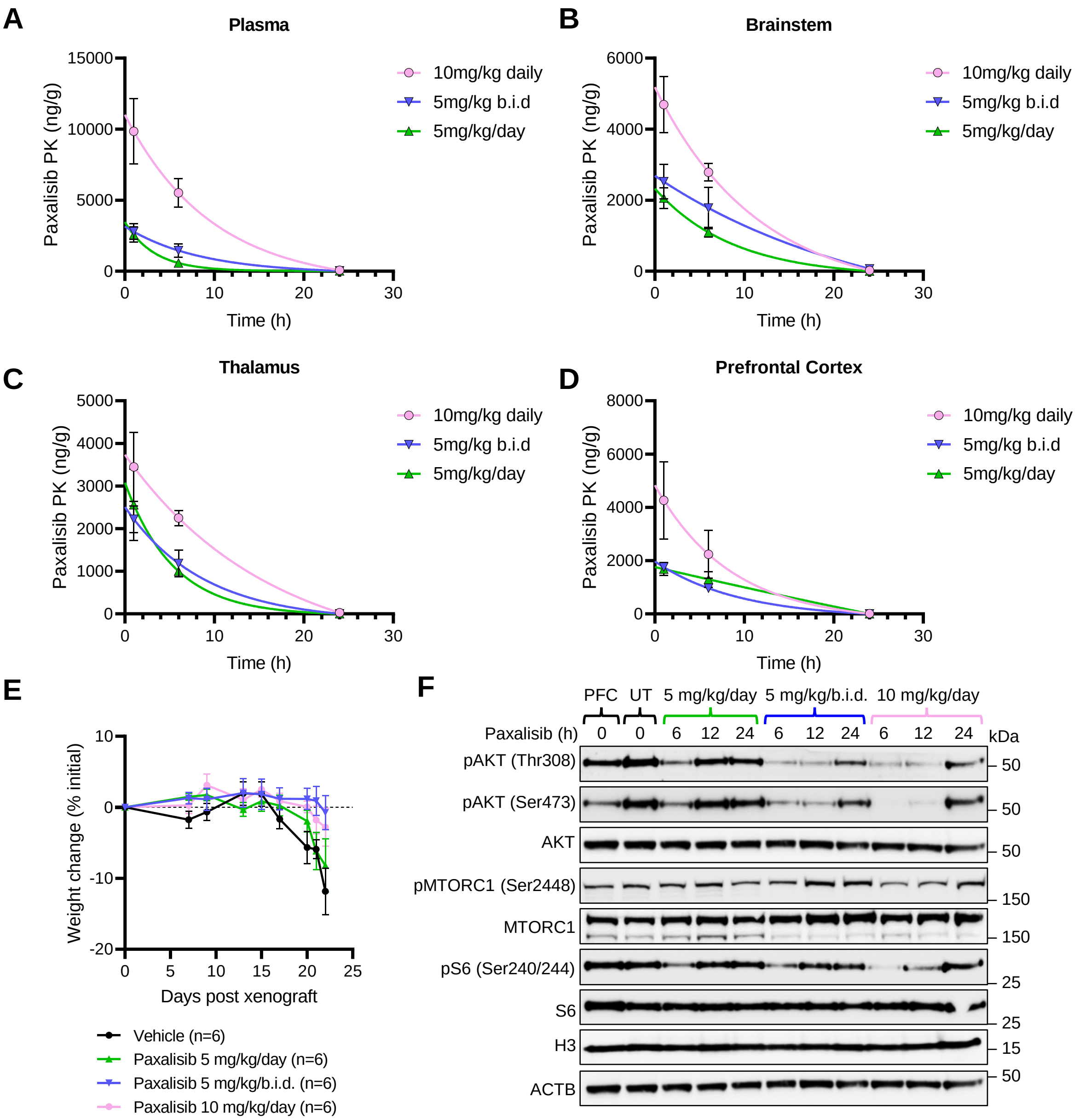

Supplementary Figure S3: Paxalisib dose optimization maintains inhibition of PI3K/Akt/mTOR signaling. (A-D) Concentration of paxalisib in plasma (A), brainstem (B), thalamus (C) or prefrontal cortex (D) after treatment with 5 mg/kg/day, 5 mg/kg/b.i.d., or 10 mg/kg/day and measured by multiple reaction monitoring mass spectroscopy (MRM). (E) SU-DIPG-XIII-P* bearing xenograft mice weights were measured after treated with either vehicle, 5 mg/kg/day, 5 mg/kg/b.i.d., or 10 mg/kg/day treatment regime over time. (F) Tumors were resected from mice treated with vehicle, 6, 12 and 24 h, post treatment after 2 weeks of paxalisib treatment (vehicle, 5mg/kg/day, 5mg/kg/b.i.d., or 10mg/kg/day) and phosphorylation and expression of key PI3K/Akt/mTOR related signaling proteins measured by immunoblotting (n=3, representative immunoblots presented).

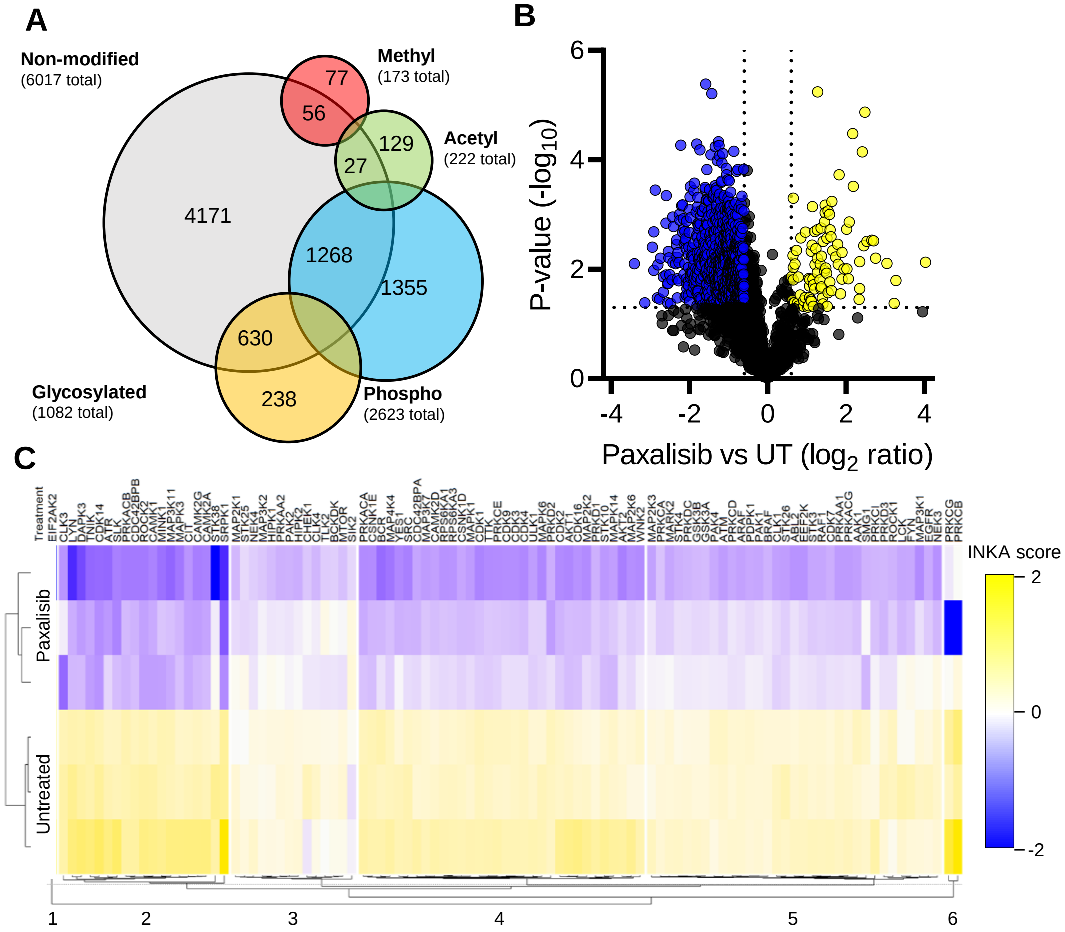

Supplementary Figure S4: Phosphoproteomic profiling of paxalisib treated DIPG models. (A) Phosphoproteomic profiling of SU-DIPG-XXXVI cells treated with 1 µM paxalisib for 6 h (n=3, biological triplicate). (B) Volcano plot of differentially phosphorylated proteins, (p<0.05 log2 fold change <-0.6 and >0.6, n=3). (C) Integrated kinase expression analysis using Integrative inferred kinase activity (INKA) analysis of paxalisib treated cells (n=3).

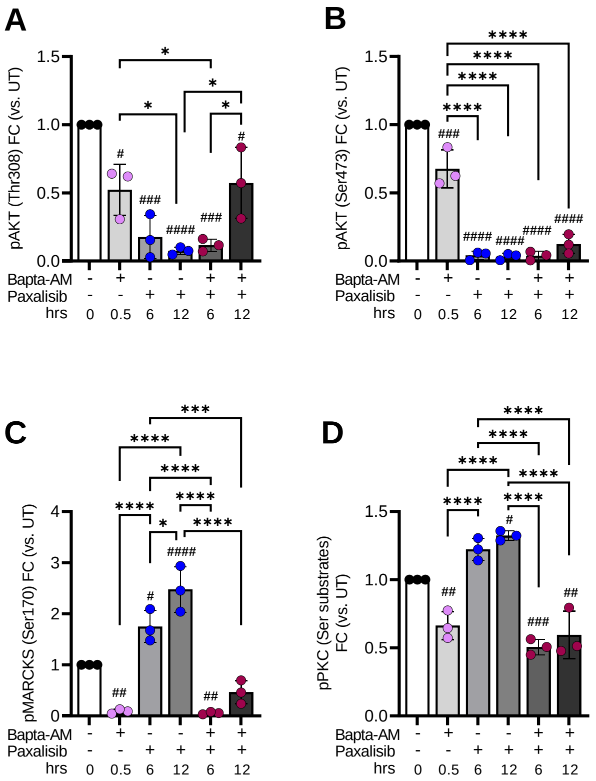

Supplementary Figure S5: Increased calcium dependent PKC following paxalisib treatment. Quantification of immunoblot measurement of proteins isolated from SU-DIPG-XIII cells treated with paxalisib or the calcium chelator BAPTA-AM (A) pAKT (Thr308), (B) pAKT (Ser473), (C) pMARCKS (Ser170) and (D) pSerPKC Substrates (n=3, one-way ANOVA, **p<0.05, **p<0.01, ***p<0.001, ****p<0.0001*).

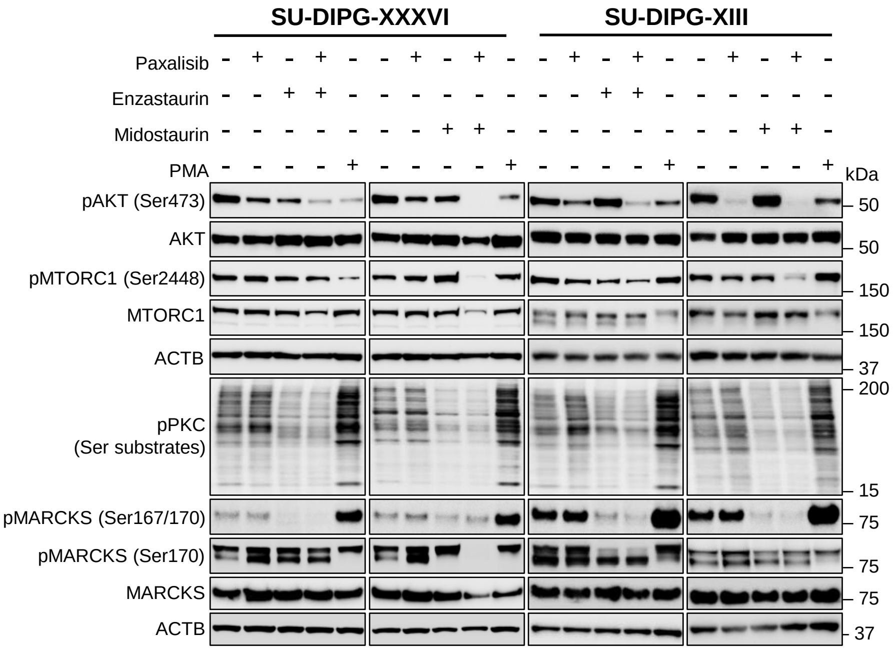

Supplementary Figure S6: Assessment of PI3K- and PKC-related signaling following the combination of paxalisib with PKC inhibitors. Protein expression and phosphorylation of key PI3K/Akt/mTOR and PKC signaling proteins was assessed after exposure to paxalisib (1 µM, 24 h), enzastaurin (5 µM, 24 h), midostaurin (5 µM, 24 h) and the PKC activator, PMA (1 µM, 24 h) (n=3, biological replicates, representative immunoblots presented).

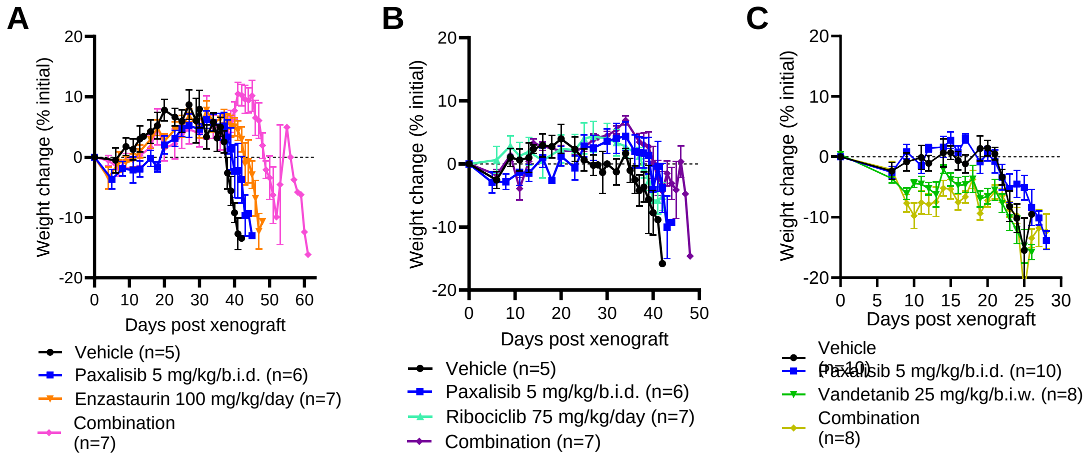

Supplementary Figure S7: DIPG xenograft mouse weight following treatment with paxalisib alone and in combination with FDA approved therapies predicted to synergize by phosphoproteomic profiling. Mouse weights recorded for SU-DIPG-XIII-P* xenograft models, following treatment with vehicle, paxalisib (5 mg/kg/b.i.d.) alone and combined with (A) enzastaurin (100 mg/kg/day), (B) ribociclib (75 mg/kg/day) or (C) vandetanib (25 mg/kg/b.i.w.).

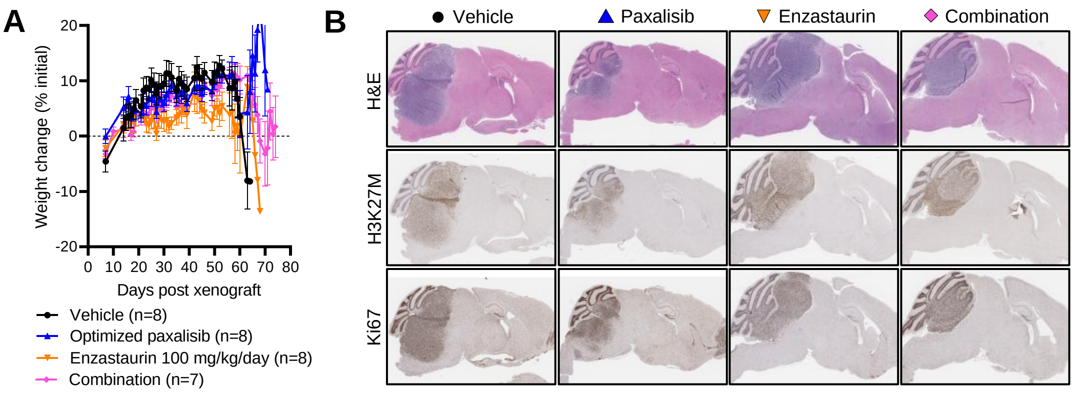

Supplementary Figure S8: RA-055 xenograft mouse model. (A) RA-055 xenograft mouse model weights, following treatment with vehicle, optimized paxalisib (5 mg/kg/b.i.d., + 175 mg/kg/day metformin) in combination with enzastaurin (100 mg/kg/day). (B) Representative immunohistochemistry for mice treated with vehicle, optimized paxalisib, in combination with enzastaurin at end stage disease.

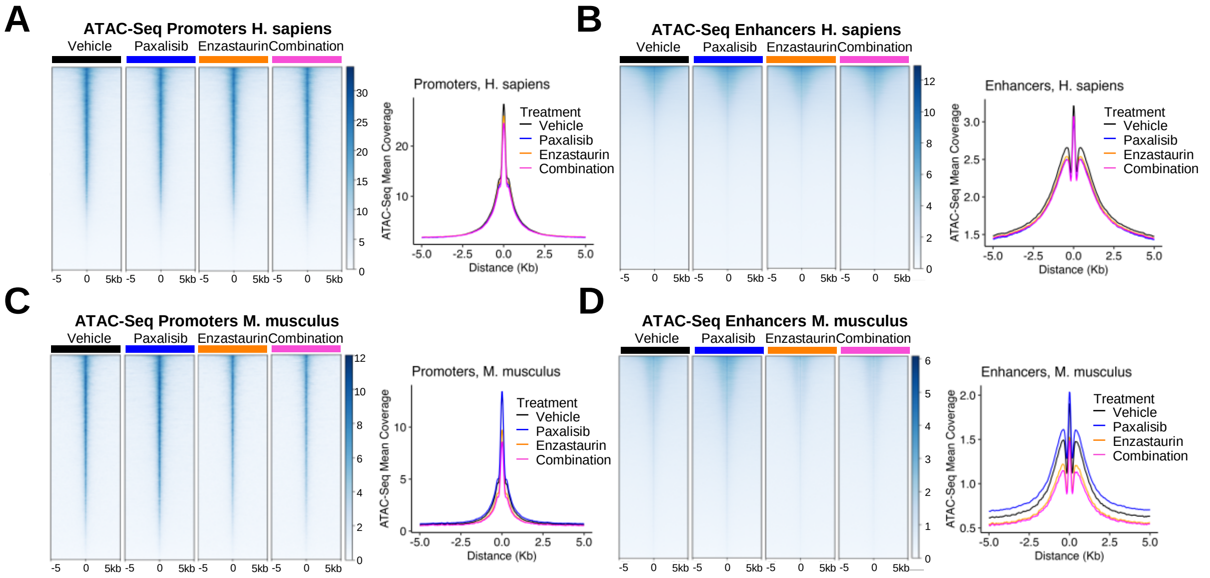

Supplementary Figure S9: Spatial transcriptomics and ATAC-seq of DIPG xenograft mouse brains treated with optimized paxalisib, enzastaurin or the combination to assess mechanisms of plasticity on treatment. (A-B) Peak maps of human ATAC-Seq (A) promoters and (B) enhancers following treatment with paxalisib, enzastaurin or the combination. (C-D) Peak maps of mouse ATAC-Seq (C) promoters and (D) enhancers following treatment with paxalisib, enzastaurin or the combination.

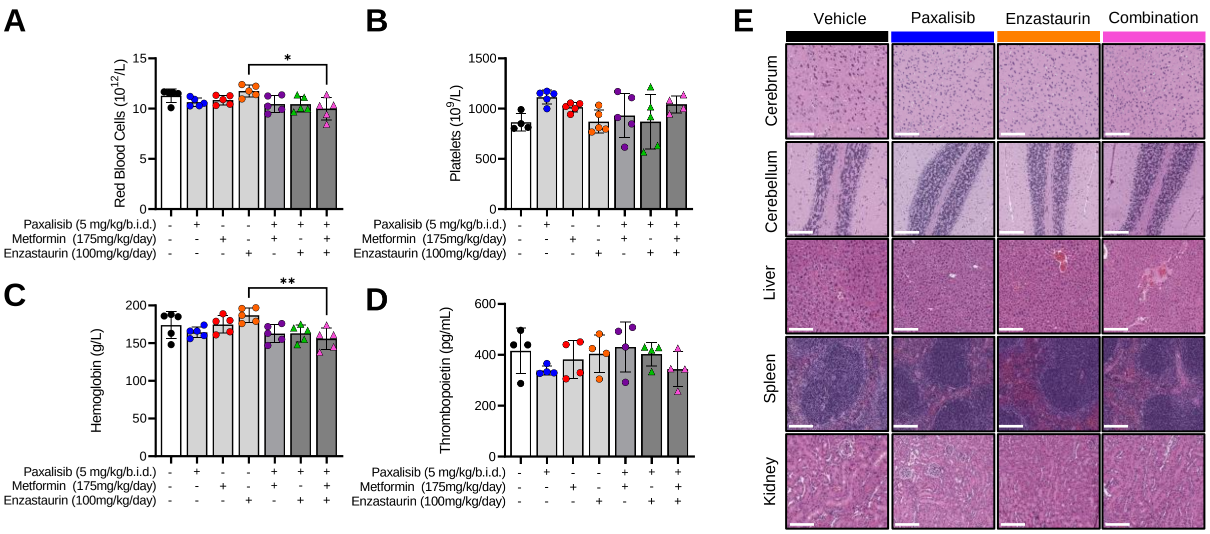

Supplementary Figure S10: Biochemical and histological analysis of syngeneic mouse model after treatment. PPK xenografts in C57BL/6J mice were treated for 4 weeks continuously with the optimized combination of paxalisib (5 mg/kg/b.i.d.) + metformin (175 mg/kg/day) and enzastaurin (100 mg/kg/day). (A-D) Red blood cell, platelet, hemoglobin and thrombopoietin counts following treatment (n=5, one-way ANOVA, *$p<0.05$, **$p<0.01$*). (E) Organs were collected from mice following 4 weeks of treatment and hematoxylin and eosin staining was used to visualize organ morphology.
