# Supplementary material for: PI3K/mTOR is a therapeutically targetable genetic dependency in diffuse intrinsic pontine glioma
Source: J Clin Invest. 2024 Feb 6;134(6):e170329. doi: 10.1172/JCI170329 (PMC10940093; doi:10.1172/JCI170329)

Full unedited blots for Figure 3

Fig. 5F

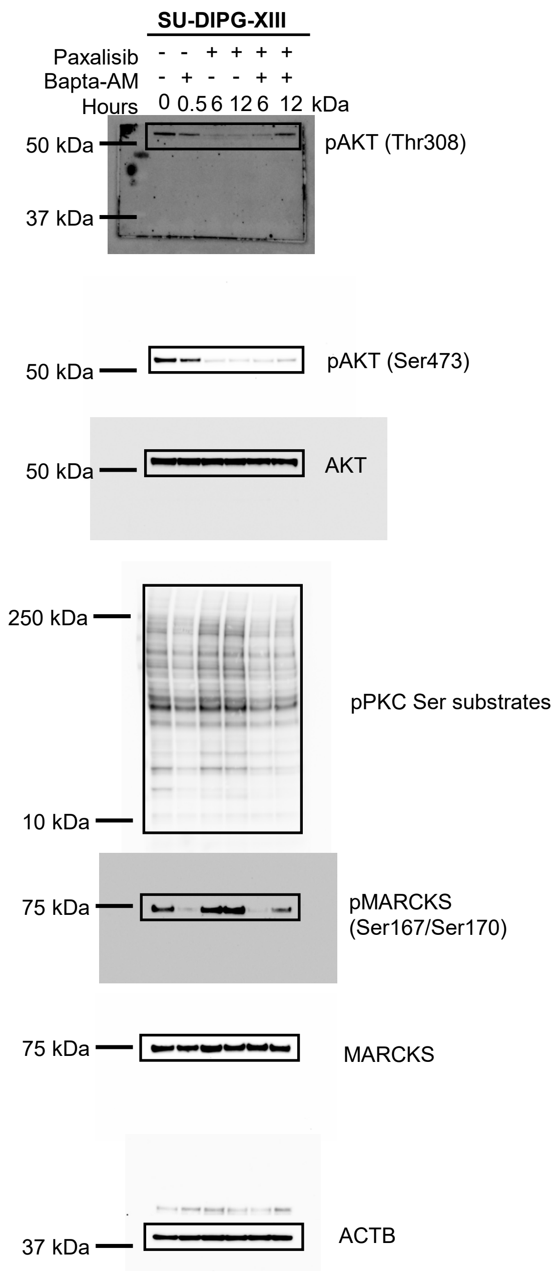

Full unedited blots for Figure 5

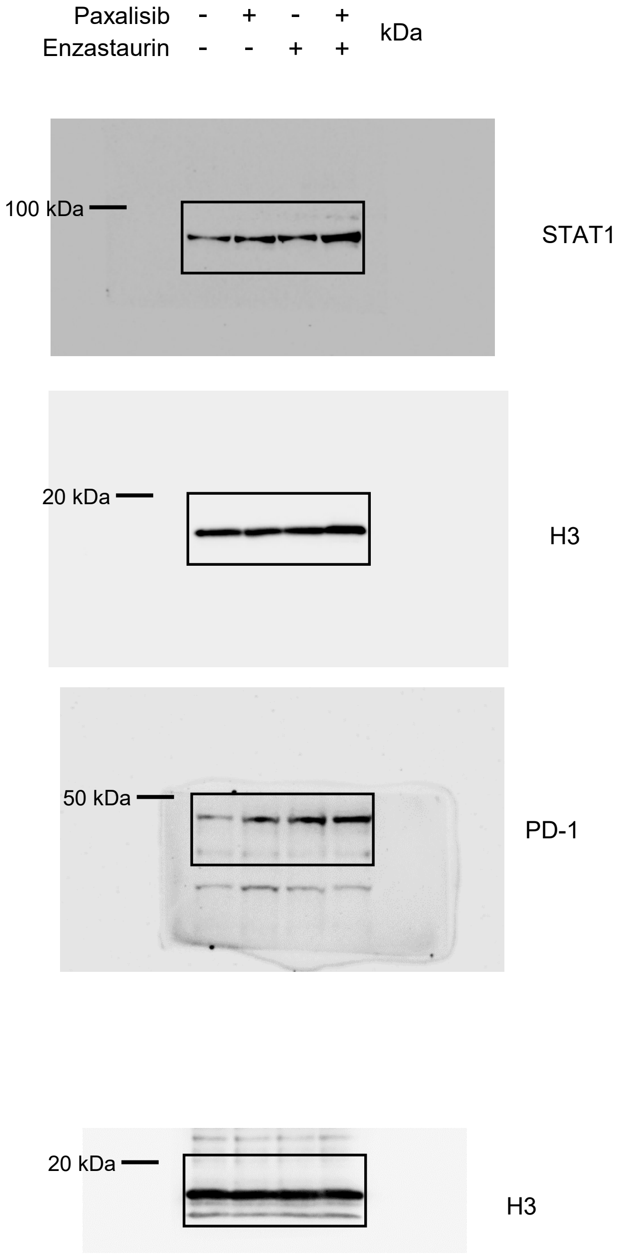

Full unedited blots for Figure S1

Fig. S1A

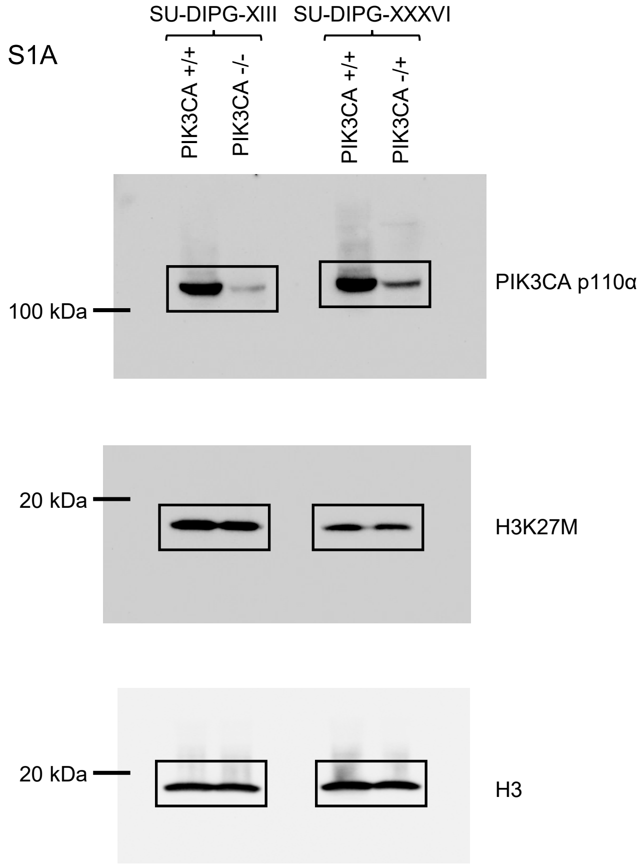

Full unedited blots for Figure S1

Fig. S1B

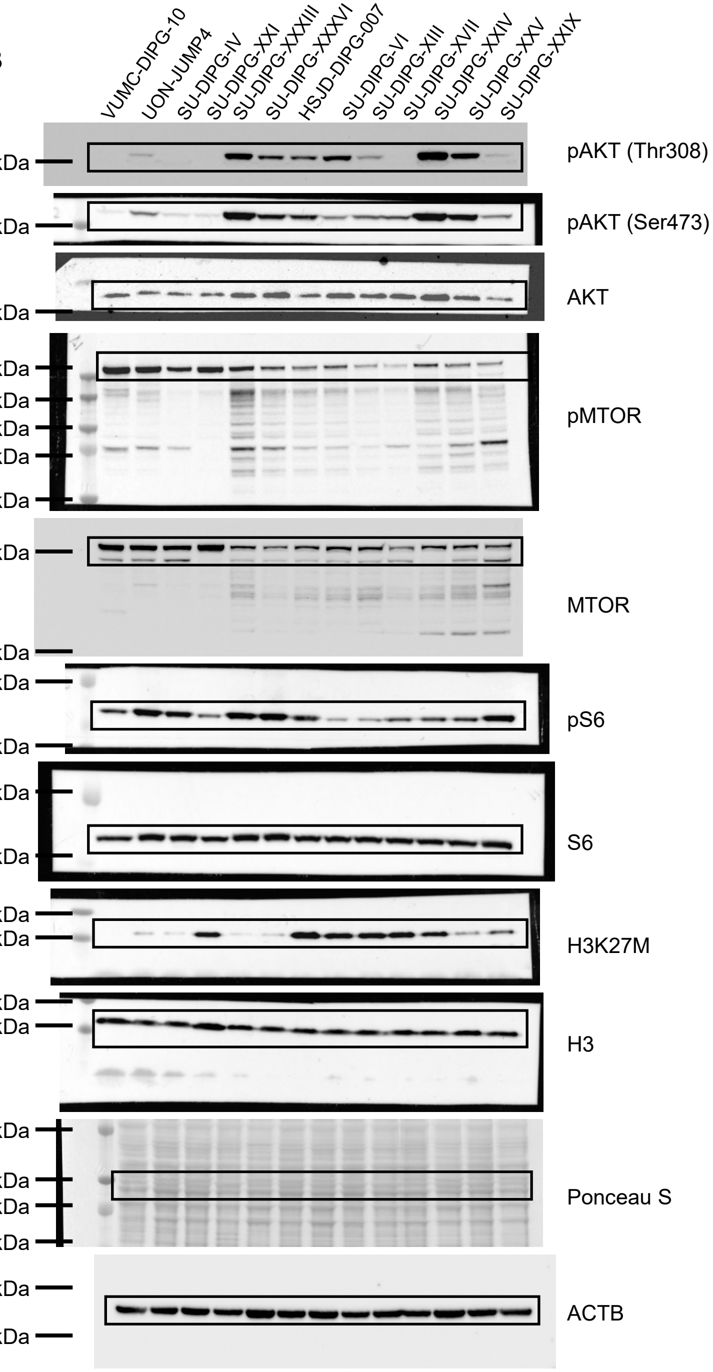

Fig. S1D

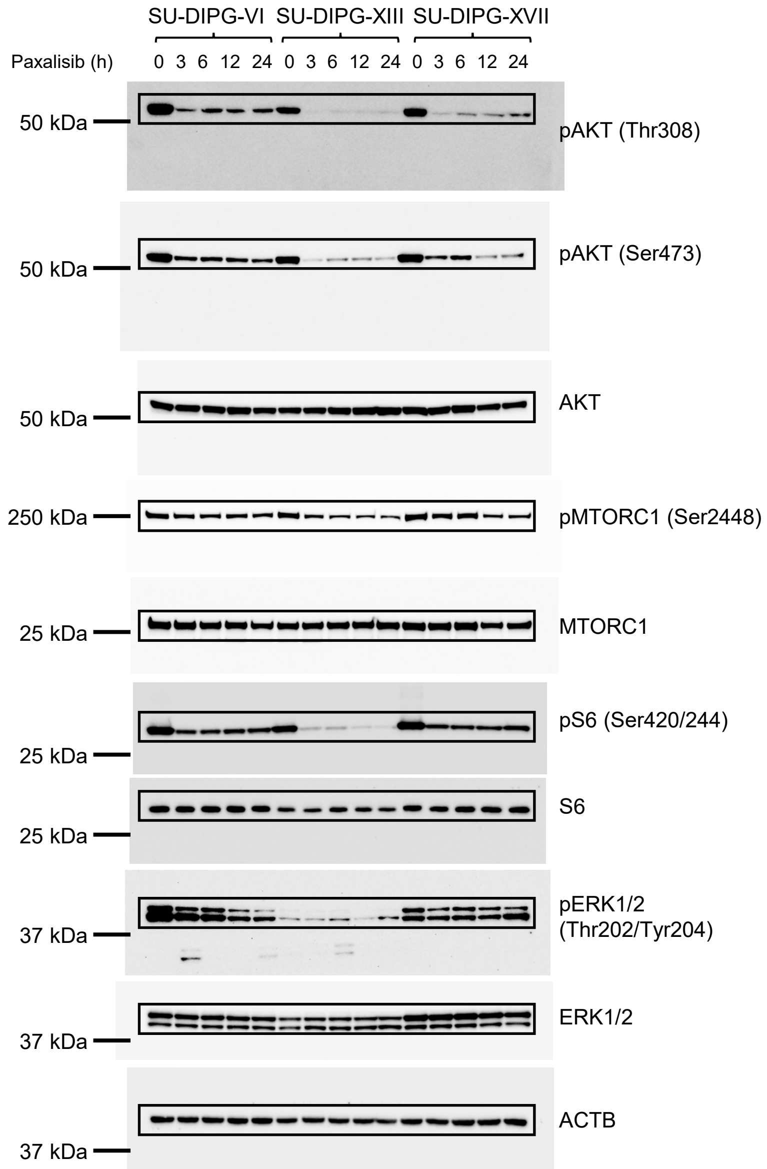

Full unedited blots for Figure S2

Fig. S2B

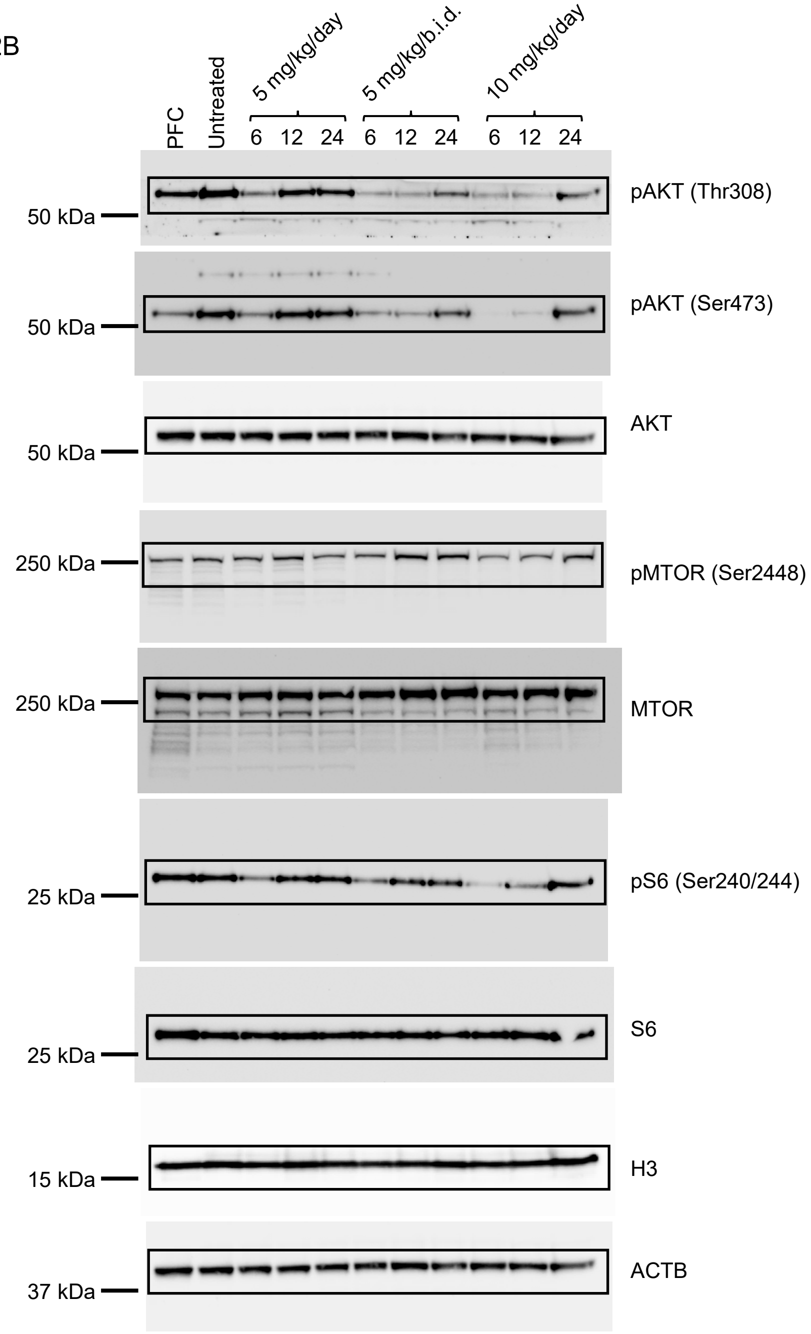

Full unedited blots for Figure S3

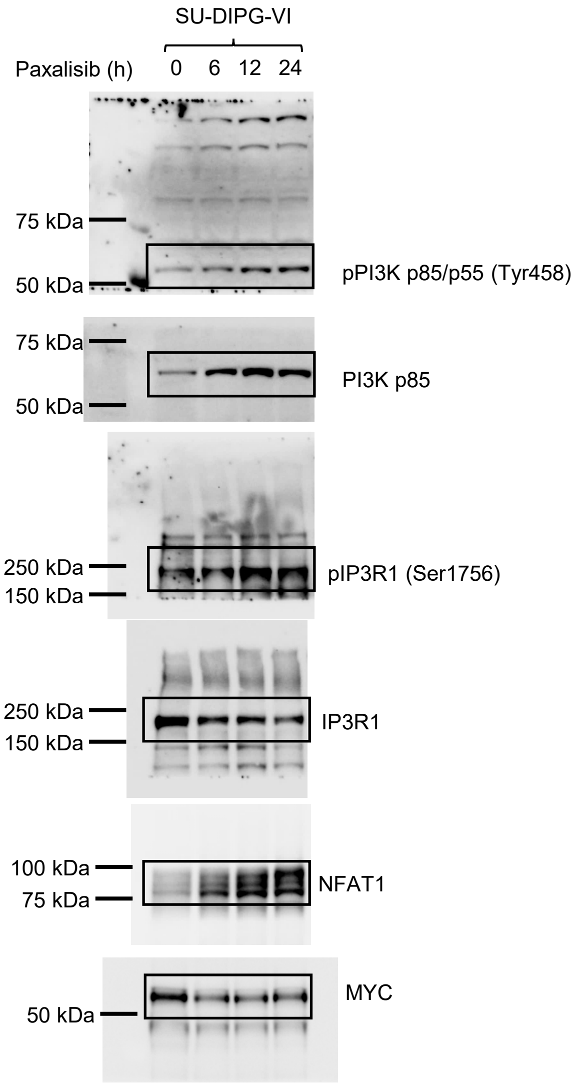

Full unedited blots for Figure S6

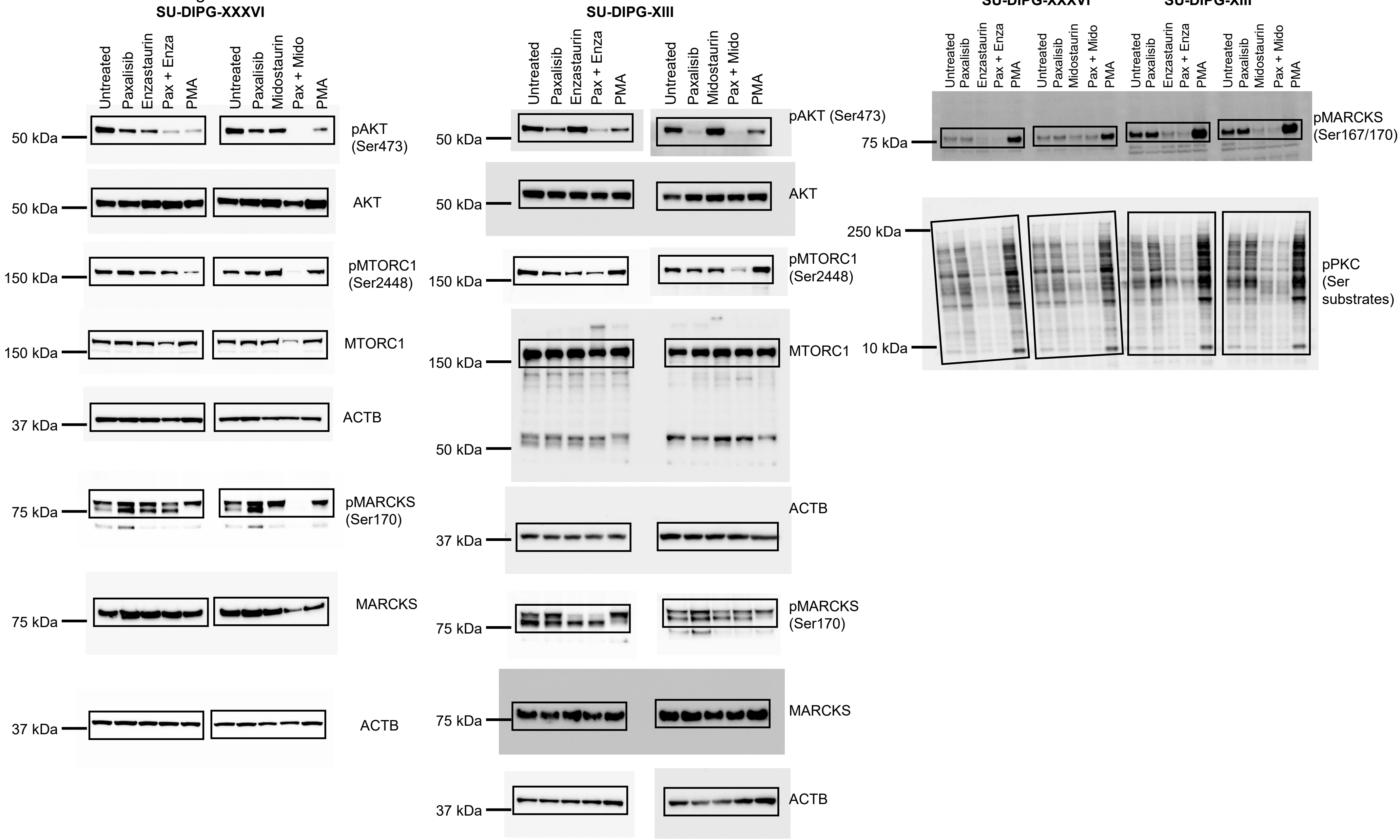

Supplement: Unedited blot and gel images [file jci-134-170329-s124.pdf]
